# Natural variation in the *Tn1a* promoter regulates tillering in rice

**DOI:** 10.1111/pbi.14453

**Published:** 2024-08-27

**Authors:** Tao Yang, Xiaoqian Ma, Quan Zhang, Lin Li, Rui Zhu, An Zeng, Wanying Liu, Haixia Liu, Yulong Wang, Shichen Han, Najeeb Ullah Khan, Jinjie Li, Zichao Li, Zhanying Zhang, Hongliang Zhang

**Affiliations:** ^1^ Frontiers Science Center for Molecular Design Breeding, Key Laboratory of Crop Heterosis and Utilization (MOE), Beijing Key Laboratory of Crop Genetic Improvement College of Agronomy and Biotechnology, China Agricultural University Beijing China; ^2^ College of Agriculture, Henan University of Science and Technology Luoyang China; ^3^ Sanya Institute of China Agricultural University Sanya China; ^4^ Sanya Nanfan Research Institute of Hainan University Sanya China

**Keywords:** tiller number, natural variation, haplotype, geographic adaptation

## Abstract

Rice tillering is an important agronomic trait that influences plant architecture and ultimately affects yield. This can be genetically improved by mining favourable variations in genes associated with tillering. Based on a previous study on dynamic tiller number, we cloned the gene *Tiller number 1a* (*Tn1a*), which encodes a membrane‐localised protein containing the C2 domain that negatively regulates tillering in rice. A 272 bp insertion/deletion at 387 bp upstream of the start codon in the *Tn1a* promoter confers a differential transcriptional response and results in a change in tiller number. Moreover, the TCP family transcription factors Tb2 and TCP21 repress the *Tn1a* promoter activity by binding to the TCP recognition site within the 272 bp indel. In addition, we identified that *Tn1a* may affect the intracellular K^+^ content by interacting with a cation‐chloride cotransporter (OsCCC1), thereby affecting the expression of downstream tillering‐related genes. The *Tn1a* ^*+272 bp*^ allele, associated with high tillering, might have been preferably preserved in rice varieties in potassium‐poor regions during domestication. The discovery of *Tn1a* is of great significance for further elucidating the genetic basis of tillering characteristics in rice and provides a new and favourable allele for promoting the geographic adaptation of rice to soil potassium.

## Introduction

Rice (*Oryza sativa* L.) is a major food crops that feeds more than half of the world's population (Khush, 1997). Breeding semi‐dwarfing varieties, also known as the Green Revolution varieties, have reduced plant height and are more resistant to lodging when high amounts of nitrogen fertiliser are applied, thus dramatically increasing rice yields (Khush, 2001). In rice, a lower number of ineffective tillers is a primary characteristic of ideal plant architecture (Khush, 1995; Peng *et al*., 2008). Therefore, clarifying the regulatory mechanisms of tiller development in rice is important for improving rice yield.

Rice tiller initiation and development are regulated by a complex network of genetic, hormonal and environmental factors (Wang *et al*., 2018a). *MONOCULM 1* (*MOC1*), the first key regulator of tiller number in rice, controls the formation of leaf axillary meristems (AM) and promotes the outward growth of axillary buds (Li *et al*., 2003). Recent studies have revealed that loss‐of‐function mutants of *MONOCULM 2* (*MOC2*) and *MONOCULM 3/ORTHOLOG OF WUSCHEL* (*MOC3/OsWUS*) exhibit a phenotype similar to *moc1* (Koumoto *et al*., 2013; Lu *et al*., 2015). Furthermore, MOC1 can act as a coactivator of MOC3, facilitating the direct binding of MOC3 to the *FLORAL ORGAN NUMBER 1* (*FON1*) promoter and activating its expression. The loss‐of‐function mutant *fon1* exhibits normal bud formation but defective outward bud elongation, leading to a reduction in tiller number (Shao *et al*., 2019). *LAX PANICLE 1* (*LAX1*) and *LAX PANICLE 2* (*LAX2*) have been identified as key players in the maintenance of AM development. *LAX1* encodes a transcription factor containing a bHLH domain, whereas LAX2 is localised in the nucleus. *LAX1* and *LAX2* regulate the initiation of rice tiller buds and contribute to the overall tillering process (Tabuchi *et al*., 2011). *TEOSINTE BRANCHED 1* (*OsTB1*) encodes a transcription factor in the TCP family that inhibits the growth of axillary buds and negatively regulates rice tiller number (Takeda *et al*., 2003). Teosinte branched 2 (OsTb2) is highly similar in sequence to OsTb1 and can interact with OsTb1 to counteract its inhibitory effect of OsTb1 on tillering via the OsMADS57‐D14 pathway, thereby promoting tiller growth (Lyu *et al*., 2020). Another gene, *OsTCP21*, was found to play a positive regulatory role in both tiller bud length and number (Wang *et al*., 2021).

Plant hormones are important determinants of rice tiller development. Tiller bud elongation is inhibited by auxin (IAA) but stimulated by cytokinin (CK) (Barbier *et al*., 2019). *DULL NITROGEN RESPONSE 1* (*DNR1*) is homologous to Arabidopsis *VAS1*, which encodes an aminotransferase that catalyses the conversion of indole‐3‐pyruvate to l‐tryptophan, thereby antagonising IAA biosynthesis. Compared to NIL‐*DNR1* ^HJX74^, NIL‐*DNR1* ^IRAP9^ showed semi‐dwarf characteristics, increased tiller number, and reduced branch number, grain number, and rice grain yield (Zhang *et al*., 2021a). A knockdown mutant of *OsIAA6* can inhibit the growth of tiller buds through positive regulation of *OsPIN1* and *OsTB1*, thereby suppressing the number of tillers in rice (Jung *et al*., 2015). *OsCKX9*, a member of the cytokinin oxidation/dehydrogenase (CKX) enzyme family, acts in the growth and development of rice basal internodes, tillers, leaves, spikelets and seeds, and the double mutant *osckx4*‐*osckx9* has a significantly increased tiller number with smaller root systems and spikes (Duan *et al*., 2009). In addition, the *osckx11* mutant significantly increased the tiller number by increasing cytokinin levels and decreasing ABA levels (Zhang *et al*., 2021b). Numerous studies have shown that mutants defective in both Strigolactone (SL) synthesis and signalling pathways exhibit more branching than the wild type (Arite *et al*., 2007, 2009; Jiang *et al*., 2013; Zhou *et al*., 2013). In addition, gibberellins (GA) are capable of inhibiting plant branching with the opposite Brassinosteroid (BR) effects (Lo *et al*., 2008; Tong *et al*., 2009). *OsKS1* encodes ent‐kaurene synthase (KS), a key enzyme involved in gibberellin biosynthesis. *OsKS1* mutants exhibit severe dwarfism and failure to flower due to blocked GA biosynthesis (Ueguchi‐Tanaka *et al*., 2005, 2007). The external application of gibberellin induces the expression of *OsGRF3* and *OsGRF10* in rice, significantly reducing tillering (Kuijt *et al*., 2014). *DLT* encodes a GRAS family protein involved in the feedback inhibition of the BR biosynthesis pathway. Exogenous application of BR reduces the expression of *DLT*, whereas *BRASSINAZOLE RESISTANT 1* (*BZR1*) inhibits the expression of *DLT*, suggesting that BR may be involved in the regulation of rice tillering by regulating *DLT* expression (Tong *et al*., 2009, 2012). Recent studies have shown that SL and BR control rice tillering by regulating the stability of the D53‐BZR1 complex and inhibiting the expression of the early responsive gene *FC1*, which is a local inhibitor of tillering (Fang *et al*., 2020). Furthermore, *OsHOX12* is important for SL‐induced *Oryza sativa 9‐CIS‐EPOXYCAROTENOID DIOXYGENASE 1* (*OsNCED1*) expression and thus mediates the process of SL regulation of tillering via ABA (Kumar *et al*., 2021; Liu *et al*., 2020).

Environmental factors also affect tillering and yield in rice. PHYTOCHROME‐INTERACTING FACTOR‐LIKE (PIL) is a transcription factor that regulates plant photomorphogenesis. OsPIL11 interacts with OsSPL14 and represses rice tillering by directly activating the *OsTB1* gene (Zhang *et al*., 2022b). Nitrogen has long been reported to be highly effective at promoting tillering in rice. *NITROGEN‐MEDIATED TILLER GROWTH RESPONSE 5* (*NGR5*) and *OsTCP19* are positive regulators of tiller number and nitrogen use efficiency (NUE) (Liu *et al*., 2021; Wu *et al*., 2020). *NITRATE‐TRANSPORTER 1.1B* (*NRT1.1B*) expression is also induced by nitrate and exhibits variation in *japonica* and *indica* rice, causing a significant increase in tiller number and yield in NILs containing *NRT1.1B* (Hu *et al*., 2015). Phosphate transporter proteins regulate phosphorus uptake and transport in rice. Inhibition of *Oryza sativa Phosphate transporter 1;4* (*OsPht1;4*) expression caused a decrease in Pi content in the stalks and, consequently, in tiller number (Ye *et al*., 2015). *Oryza sativa* CATION‐CHLORIDE COTRANSPORTER 1 (OsCCC1) is a K^+^/Cl^−^ cotransporter protein. Compared with the wild type, the RNAi lines had reduced K^+^ and Cl^−^ contents, but no change in Na^+^ content, and caused a decrease in yield, with a significant reduction in tiller number, 1000‐grain weight, and grain numbers per panicle (Chen *et al*., 2016). *Oryza sativa HIGH‐AFFINITY POTASSIUM TRANSPORTER* (*OsHAK18*) mediates phloem K^+^ loading and redistribution, and its knockout mutants have fewer tillers (Shen *et al*., 2023). *OsHAK5* overexpression increased polar auxin transport (PAT), tiller number, and root hair length (Yang *et al*., 2020). Although K^+^ channel proteins are involved in tillering regulation, the mechanism through which K^+^ regulates tillering in rice remains unclear.

The C2 domain is a Ca^2+^‐dependent membrane‐targeting domain that plays an important role in abiotic and biotic stresses tolerance and membrane targeting in plants (Zhang *et al*., 2022a). In *Arabidopsis*, QKY contains four conserved C2 domains that are involved in the growth of floral meristematic tissues and root hair (Vaddepalli *et al*., 2014). In rice, a novel C2 domain‐containing protein (OsC2DP) is involved in the positive regulation of salt tolerance (Fu *et al*., 2019). Recent studies have shown that numerous OsC2DPs are involved in phytohormones‐related responses in rice (Zhang *et al*., 2022a). However, the specific functions of OsC2DPs in regulating tiller numbers remain unclear.

Most of the previously mentioned cloned tiller‐regulating genes have been obtained using mutants or reverse genetics, and only a few favourable allelic variants have been unearthed from natural populations. At the present stage, genome‐wide association studies (GWAS) have been proven to be an ideal tool for identifying more loci associated with complex traits in the germplasm with diverse natural variations (Huang *et al*., 2010; Li *et al*., 2018; Liu *et al*., 2021; Yu *et al*., 2018).

Our previous studies on dynamic tillering showed that genes with a pattern of steady transition from tiller to panicle development (ST‐TtP) and expression pattern 2 (R24‐P2) in roots at 24:00 were more likely to be involved in the regulation of tillering in rice (Ma *et al*., 2020). In this study, we reported that *Tn1a* negatively regulates tiller number. Deletion of 272 bp increased *Tn1a* expression and decreased tiller number. The inhibitory effect of 272 bp on expression may be due to the direct binding by Tb2 and TCP21. Tn1a interaction with OsCCC1 alters K^+^ content and may have led to changes in the expression of tillering‐related genes. The geographic distribution of the *Tn1a* allele was closely correlated with the soil K^+^ content. The discovery of *Tn1a* enriches our understanding of the regulatory network of rice tillering and provides a favourable variant for rice adaptation to potassium‐deficient soil environments.

## Results

### *Tn1a* negatively regulates rice tillering

**Figure 1:**
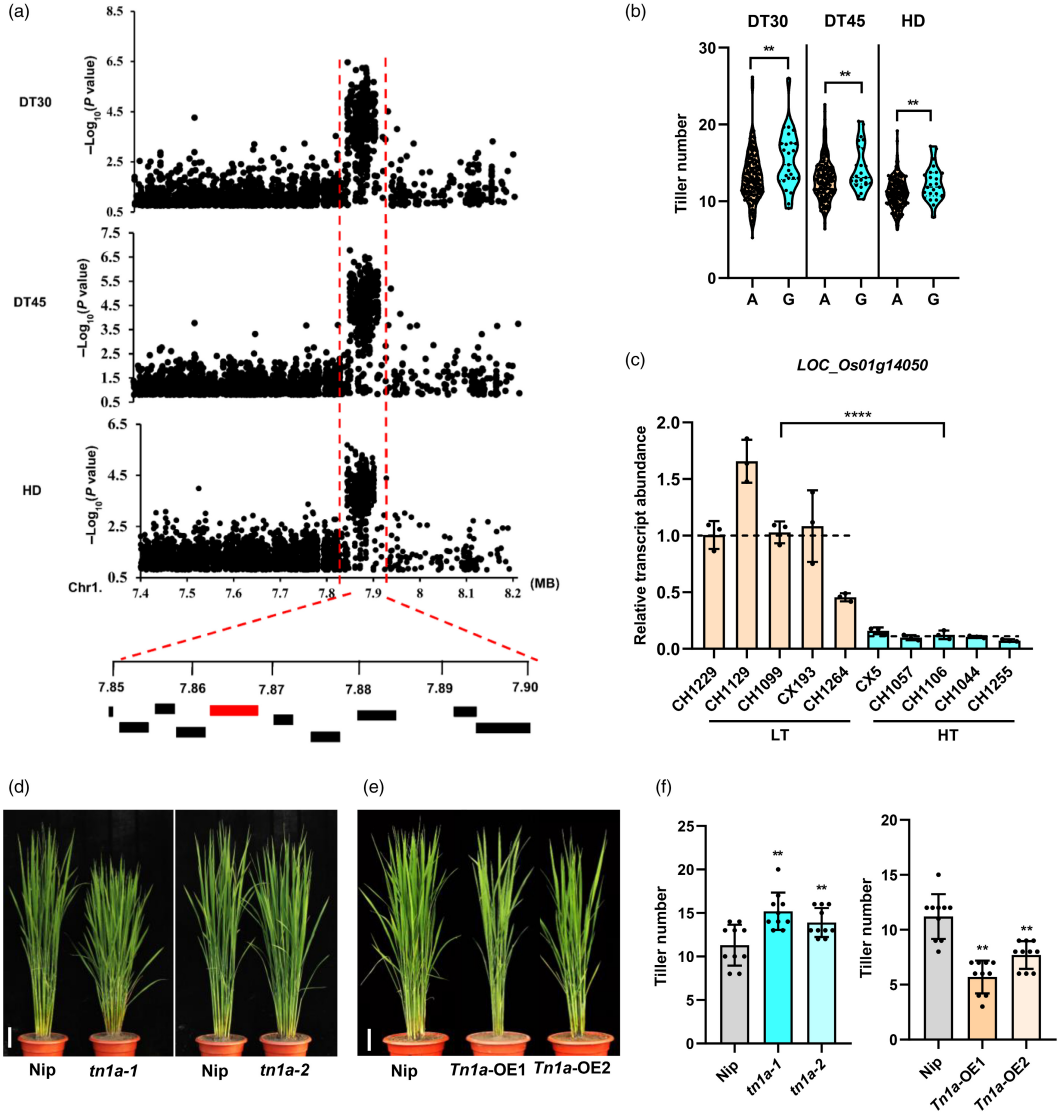
*Tn1a* confers natural variation in rice tillering. (a) The genome‐wide association signals for tiller number at DT30, DT45 and HD stages are shown in the region at 7.84–7.92 Mb region on chromosome 1 (*x* axis). −log_10_ (*P* value) from the linear model, plotted on the *y* axis. The ORFs included in the candidate region are labelled. *Tn1a* is indicated in red. (b) Analysis of tiller number for *indica* varieties stratified by genotype at Chr1_7844142. Statistical significance was determined using a two‐tailed *t*‐test; (*n* = 165 for genotype A; *n* = 25 for genotype G). (c) Expression analysis of *Tn1a* in tiller base of LT and HT varieties (LT represents low‐tiller varieties, HT represents high‐tiller varieties). Data represent mean ± SD (*n* = 3). (d) and (e) Plant architecture of Nip, *tn1a‐1*, *tn1a‐2*, *Tn1a*‐OE1 and *Tn1a*‐OE2 lines at the tillering stage. Scale bar = 10 cm. (f) Tiller numbers of Nip, *tn1a‐1*, *tn1a‐2*, *Tn1a*‐OE1 and *Tn1a*‐OE2 lines. Data are presented as mean ± SD (*n* = 10). Statistical significance in (b, c, f, g) was determined using a two‐sided *t*‐test (**P < 0.01, ****P < 0.0001).

To identify the genes regulating tiller development, we previously investigated the phenotype of dynamic changes in the tiller number of 377 rice varieties and performed genome‐wide association studies (Ma *et al*., 2020). Among the identified loci, a novel locus *qTn1.7* (7.84–7.92 Mb) on chromosome 1 was repeatedly detected with strong signals at the 30th day, 45th day and heading stages (DT30, DT45, and HD) (Figure 1a). Variation in the leading SNP Chr1_7844142 (G to A), was associated with higher and lower tiller numbers at different tillering stages (Figure 1b). Given the estimated LD decay rate of approximately 120–170 kb in landraces (Huang *et al*., 2010; Mather *et al*., 2007), we analysed 10 annotated candidate genes in this QTL (Figure 1a, Table S1). Analysis of SNPs that could be significantly associated at all three stages in *qTn1.7* revealed that only *LOC_Os01g14050* and *LOC_Os01g14090* had a non‐synonymous mutation in the coding region; except for *LOC_Os01g14030*, all other candidate genes showed variations in the promoter region (Table S1). We examined the expression of 10 candidate genes at the stem base of randomly selected natural *indica* varieties with high and low tiller numbers distinguished by the lead SNP (Table S2). Furthermore, we found that the expression of *LOC_Os01g14050* and *LOC_Os01g14100* was significantly higher in low‐tiller varieties (LT) than in high‐tiller varieties (HT), whereas other candidate genes showed no significant differences (Figure 1c; Figure S1). The expression level of *LOC_Os01g14050* in roots at 24:00 h during vegetative growth was significantly higher than that of *LOC_Os01g14090* and *LOC_Os01g14100* (Figure S2). Collectively, these results suggest that *LOC_Os01g14050* may be the candidate gene for *qTn1.7*, designated as *Tn1a*.

To validate the function of *Tn1a* in rice tillering, we generated the *Tn1a* mutants, *tn1a‐1* and *tn1a‐2*, using CRISPR/Cas9‐mediated gene editing in a Nipponbare (Nip) genetic background. The *tn1a‐1* and *tn1a‐2* mutants harboured 5 bp and 7 bp deletions in the third exon, respectively, resulting in frameshifts (Figure S3a). Compared with Nip, *tn1a‐1* and *tn1a‐2* plants showed significantly increased tiller number (Figure 1d,f), indicating that *Tn1a* negatively regulates tiller number. To further confirm its function, we generated both *Tn1a* overexpression lines (*Tn1a*‐OE1 and *Tn1a*‐OE2) and *Tn1a* RNAi lines (*Tn1a*‐RNAi‐1 and *Tn1a*‐RNAi‐2) in Nip background. The positive transgenic lines showed that the tiller number was significantly decreased in the overexpression lines and increased in the RNAi lines (Figure 1e,f; Figure S3b–e). In addition, we investigated the panicle phenotypes of different transgenic lines. The *tn1a‐1* and *tn1a‐2* mutants and *Tn1a*‐RNAi‐1 and *Tn1a*‐RNAi‐2 lines showed an increase in secondary branch number, grain number per panicle, and grain yield per plant, in contrast to the *Tn1a*‐OE1 and *Tn1a*‐OE2 lines, which showed a significant decrease in panicle length, primary branch number, secondary branch number, grain number per panicle, and grain yield per plant (Figure S4). Taken together, these results suggest that *Tn1a* negatively regulates rice tillering.

### A 272 bp insertion inhibits *Tn1a* expression to promote tillering

**Figure 2:**
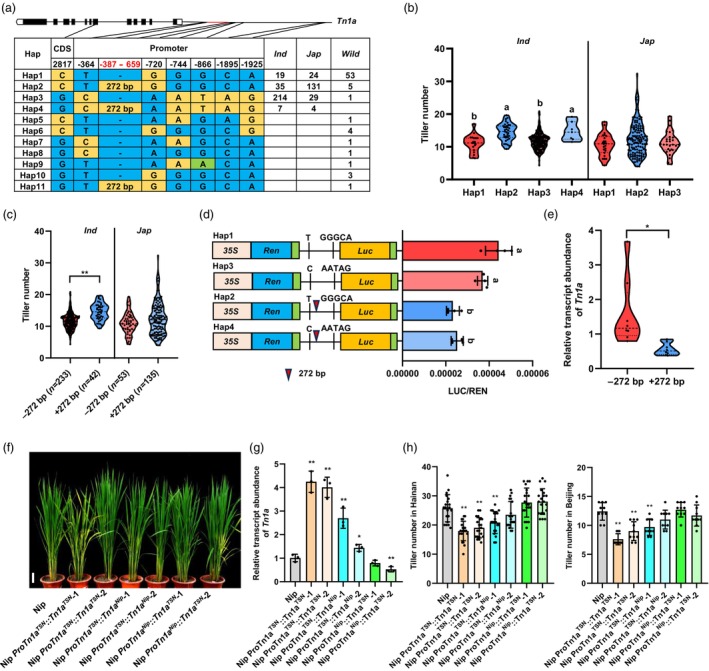
Association of 272 bp indel in the *Tn1a* promoter is associated with gene expression and tiller number in the *indica* subpopulation. (a) Natural variation and haplotype analysis of *Tn1a* in 463 Asian cultivated rice and 71 wild rice accessions. *Ind*, *indica*; *Jap*, *japonica*. (b) Violin plots for tiller number of rice varieties carrying different *Tn1a* haplotypes. All data are from plants grown under normal paddy‐field fertilisation conditions in Guangxi, China in 2018. Different letters indicate significant differences at *P* < 0.05 according to the two‐sided Student's *t*‐test. (c) Distribution of tiller number in cultivated rice grouped by the 272 bp indel. Data presented as mean ± SE (*indica* accessions: −272 bp, *n* = 233; +272 bp, *n* = 42; *japonica* accessions: −272 bp, *n* = 53; +272 bp, *n* = 135). (d) Comparison of promoter activities of four *Tn1a* haplotypes by dual luciferase assay in the *indica* subpopulation. Data are presented as mean ± SD (*n* = 4). Different letters indicate significant differences at *P* < 0.05 according to the two‐sided Student's *t*‐test. (e) Distribution of *Tn1a* expression in *indica* subpopulation grouped by the 272 bp indel. (f) Plant architecture of Nip, *ProTn1a* ^*TSN*^ *::Tn1a* ^*Nip*^, *ProTn1a* ^*Nip*^ *::Tn1a* ^*TSN*^, and *ProTn1a* ^*TSN*^ *::Tn1a* ^*TSN*^ transgenic lines at the tillering stage. Scale bar = 10 cm. (g, h) Expression analysis of *Tn1a* in Nip, *ProTn1a* ^*TSN*^ *::Tn1a* ^*Nip*^, *ProTn1a* ^*Nip*^ *::Tn1a* ^*TSN*^, and *ProTn1a* ^*TSN*^ *::Tn1a* ^*TSN*^ transgenic lines and tiller numbers in Hainan and Beijing. Values in (e) are mean ± SD (*n* = 3). Values in (h) are presented as mean ± SD (*n* = 10 in Beijing; *n* = 20 in Hainan). In (c, e, g, h), *P*‐values were determined using a two‐sided Student's *t*‐test (*P < 0.05, **P < 0.01).

Given that the significant association between *Tn1a* and tiller development, we next extracted the significant variations in previous GWAS within *Tn1a* (containing the 2‐kilobase promoter, coding sequence (CDS) and 3′ untranslated region (UTR)) from 463 cultivated varieties and 71 wild rice accessions to verify its functional locus and identified 8 variations, including 7 SNPs (with one in CDS and six in promoter) and a 272 bp indel (at 387 bp upstream of the start codon in promoter). We compared the genomic sequences corresponding to the ORF and promoter regions of *Tn1a* and found that the promoter region had high nucleotide diversity (Figure S5). And phylogenetic analysis revealed a clear differentiation of *Tn1a* between two subspecies (*indica* and *japonica*) (Figure S6). We then performed haplotype analysis using the 272 bp indel and 7 SNPs and found 11 haplotypes (Hap1–Hap11), with 10 haplotypes (Hap1–Hap3, Hap5–Hap11) present in wild rice (Figure 2a). Among 4 haplotypes in cultivated rice, Hap2 is predominantly present in the *japonica* subpopulation, whereas Hap3 is predominantly present in the *indica* subpopulation. Comparing the numbers of tillers among these 4 haplotypes in each subspecies, we found that those in Hap2 and Hap 4 are significantly higher than those in Hap1 and Hap3 in *indica*, but there is no difference between Hap1 and Hap3, and between Hap2 and Hap4 in *indica*; and there is no difference among haplotypes in the *japonica* subpopulation (Figure 2b; Figure S7a). When comparing the 8 sequence variations among these 4 haplotypes, we found that only the 272 bp indel presented variation between Hap1 and Hap2, and between Hap3 and Hap4; and at the same time only the 272 bp indel presented no variation between Hap1 and Hap3, and between Hap2 and Hap4. The tiller number is significantly higher in varieties with the 272 bp insertion (+272 bp) than those without it (−272 bp) (Figure 2c; Figure S7b). We inferred according to these results that the 272 bp indel in promotor may confer *Tn1a* the functional variability in rice tillering.

We than carried out transient expression and qPCR assay to compared the promoter activity of the four major haplotypes (Hap1–Hap4), among which, Hap2 and Hap4 contain the 272 bp segment (+272 bp), while Hap1 and Hap3 do not contain it (−272 bp). We observed lowered LUC expression when the reporter was driven by Hap2 and Hap4 (Figure 2b), and lower level of *Tn1a* expression in varieties containing the 272 bp insertion than that in varieties without the insertion (Figure 2e). These results suggest that the 272 bp insertion maybe suppress the expression of *Tn1a* and finally involve in rice tillering. To examine the distribution of 272 bp indel in natural populations, the identification of the 272 bp segment was carried out in an enlarged population with 552 germplasm varieties, and it was found that 79.5% (159/200) *japonica* varieties had a 272 bp segment, whereas in 84.3% (297/352) *indica* varieties, the segment was absent (Figure S8a,b). Collectively, these results indicate that the 272 bp indel may be the main functional differentiation of *Tn1a* between *indica* and *japonica* accessions.

To further clarify the effect of eight natural variations of *Tn1a* on tillering in rice, complementary constructs *ProTn1a* ^*TSN*^ *::Tn1a* ^*Nip*^, *ProTn1a* ^*Nip*^ *::Tn1a* ^*TSN*^, and *ProTn1a* ^*TSN*^ *::Tn1a* ^*TSN*^ constructs were transformed into Nip (Figure 2f), where TSN (*indica* variety) and Nip (*japonica* variety) present variations at loci of the 272 bp indel and the CDS (Figure S9). Two independent complementary lines were obtained for each transformation. The qPCR assay showed that the expression of *Tn1a* was higher in complementary lines carrying the TSN promoter than in those carrying the Nip promoter (Figure 2g). Moreover, when comparing tillering performance in Beijing and Hainan, the number of tillers in complementary lines carrying the TSN promoter was significantly lower than that in Nip, whereas no significant differences were observed in complementary lines carrying the Nip promoter (Figure 2h). These results indicate that the 272 bp insertion in the *Tn1a* promoter is associated with decreased gene expression, which in turn contributes to a higher tiller number.

To further investigate whether *Tn1a* is involved in the initiation or elongation of tiller development, we compared the growth status of tiller buds of different transgenic plants and observed the magnified views of the longitudinal sections of shoot apical meristems (SAMs) and axillary buds, and we found that *Tn1a* significantly repressed bud outgrowth, but failed to repress bud initiation (Figure S10a,b).

### The 272 bp insertion allele is directly inhibited by TCP21 and Tb2

**Figure 3:**
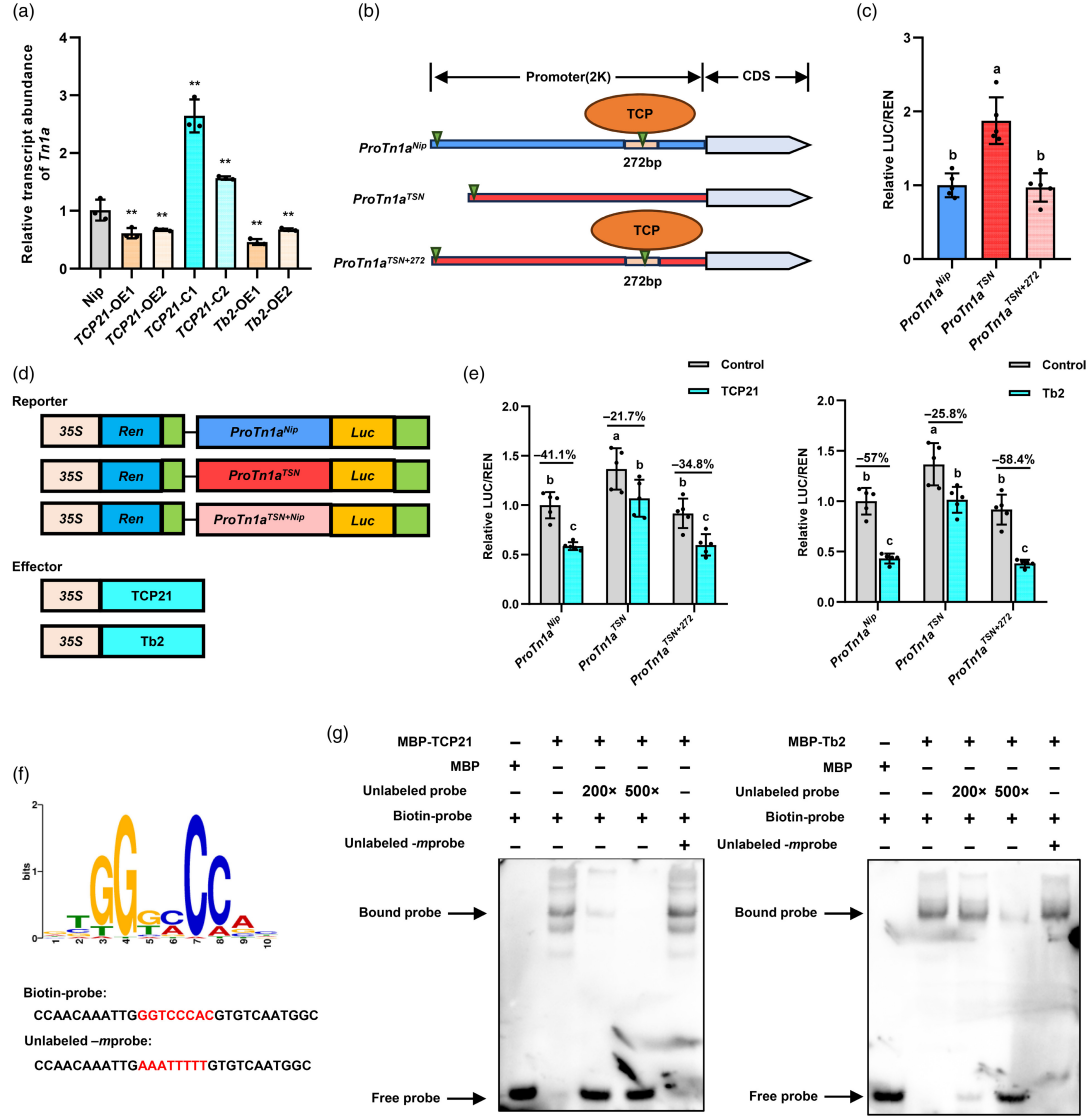
Transcriptional effects of TCP21 and Tb2 in the 272 bp insertion allele. (a) Expression analysis of *Tn1a* in stem base of *TCP21* and *Tb2* transgenic lines. *P*‐values were determined using a two‐sided Student's *t*‐test (**P < 0.01). (b) Binding sites of TCP transcription factors in *Tn1a* ^*Nip*^, *Tn1a* ^*TSN*^, or *Tn1a* ^*TSN+272*^ promoter variants. The green triangle indicates the position of binding motifs. (c) Promoter activity of *Tn1a* ^*Nip*^ or *Tn1a* ^*TSN*^ or *Tn1a* ^*TSN+272*^ promoter variants. (d) The reporter vector contained *Tn1a* ^*Nip*^, *Tn1a* ^*TSN*^ or *Tn1a* ^*TSN+272*^ promoter sequences, whereas the effector vector contained TCP21 or Tb2 CDS. (e) Transcriptional activity of TCP21 and Tb2 on 272 bp insertion. (f) Predicted binding motif in 272 bp insertion/deletion and the biotin‐probes and Unlabeled‐*m*probes. (g) EMSA assays show the binding of TCP21 and Tb2 to the 272 bp insertion. Values in (c and e) are mean ± SD from five independent experiments. Different letters indicate significant differences at *P* < 0.05 according to the two‐sided Student's *t*‐test.

Sequence analysis of the 272 bp insertion using the PlantPAN 3.0 database (Chow *et al*., 2019) revealed a TCP recognition sequence (Figure 3e). Given that TCP21 and Tb2 positively regulate rice tillering (Lyu *et al*., 2020; Wang *et al*., 2021), we investigated whether Tb2 and TCP21 directly repress *Tn1a* expression. We examined the expression levels of *Tn1a* in the stem bases of different transgenic lines of *TCP21* overexpression and knockout lines (*TCP21*‐OE1, *TCP21*‐OE2, *TCP21*‐C1 and *TCP21*‐C2), *Tb2* overexpression (*Tb2*‐OE1 and *Tb2*‐OE2) and wild‐type plants, and the results showed that Tb2 and TCP21 negatively regulate the expression of *Tn1a* (Figure 3a). Next, transient expression assays were performed using LUC fusion reporters driven by *Tn1a* ^*Nip*^, *Tn1a* ^*TSN*^ and *Tn1a* ^*TSN+272*^ promoter variants (Figure 3b). Initially, the promoter activities of the three promoter variants were tested, and it was observed that the promoter driven by *Tn1a* ^*TSN*^ showed higher promoter activity than the other promoter variants (Figure 3c). In addition, TCP21 and Tb2 transcriptionally repressed *proTn1a* ^*Nip*^, *proTn1a* ^*TSN*^, and *proTn1a* ^*TSN+272*^ in transient expression assays, and *proTn1a* ^*TSN*^ showed significantly higher LUC activation than *proTn1a* ^*Nip*^ and *proTn1a* ^*TSN+272*^ (Figure 3d,e). We then performed EMSA using the 30 bp sequence from 272 bp, which contained the TCP recognition site as the probe (Figure 3f). Unsurprisingly, recombinant MBP‐TCP21 and MBP‐Tb2 were able to bind to the labelled probe, but not to the mutated probe (Figure 3g). Based on these findings, it can be inferred that variations in the 272 bp insertion within the *Tn1a* promoter are associated with differential binding and regulation by TCP21 and Tb2.

### *Tn1a* encodes a C2‐domain protein highly expressed during tillering and booting stages

**Figure 4:**
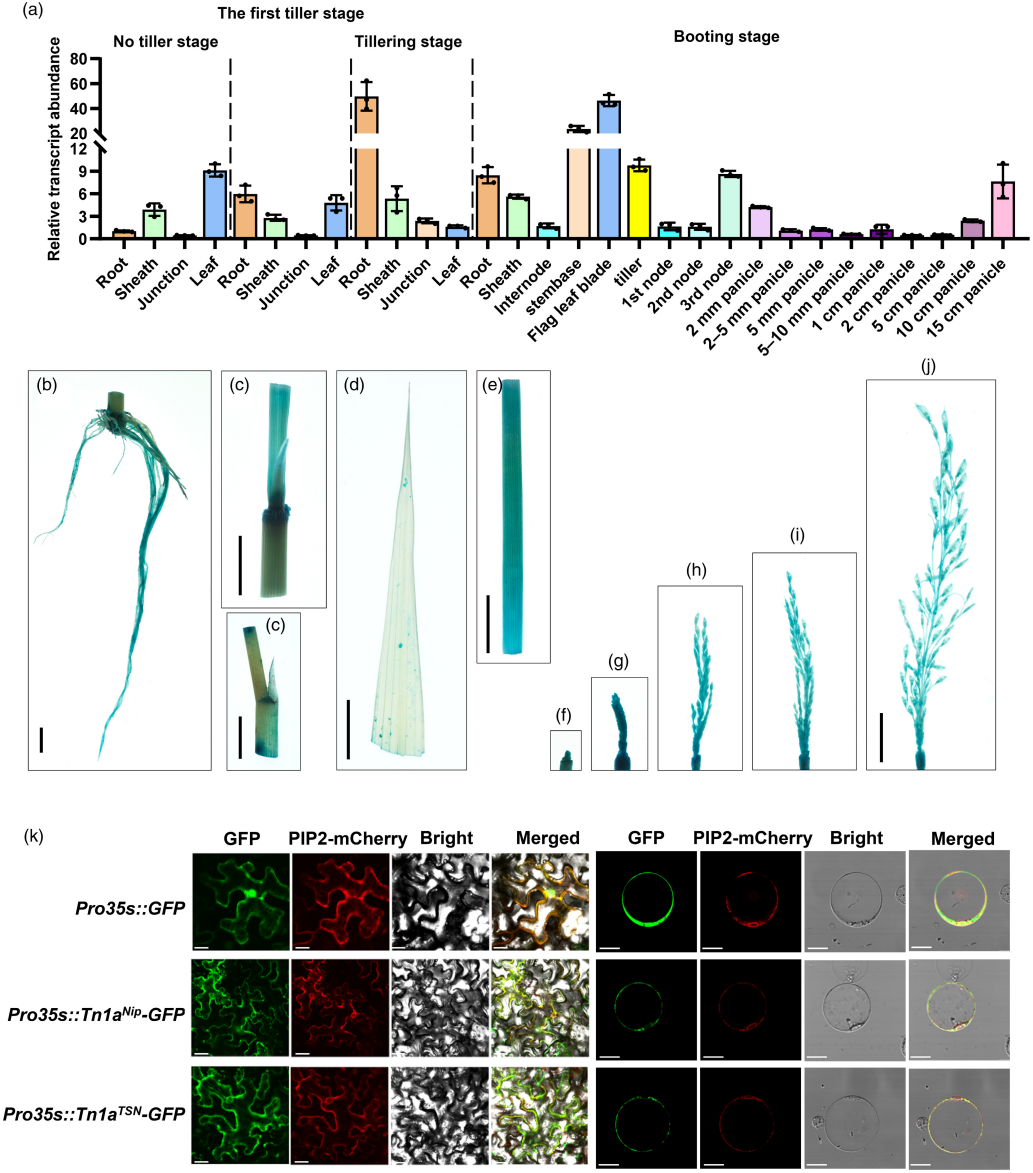
Expression pattern of *Tn1a*. (a) Relative expression of *Tn1a* in various tissues at the different growth stages. Data represent mean ± SD (*n* = 3). (b–e) GUS staining of the *ProTn1a::GUS* transgenic plants at the vegetative stage. (b) Root. (c) Internode. (d) Leaf sheath. (e) Leaf. (f–j) GUS staining of panicles of different length in *ProTn1a::GUS* transgenic plants during the reproductive stage. Scale bar = 1 cm. (k) Subcellular localisation of the Tn1a‐GFP fusion proteins of Nip and TSN in rice protoplasts and tobacco. Scale bar = 20 μm.

To further illustrate the molecular function of *Tn1a* in regulating tillering in rice, plant tissues were collected at different tiller developmental stages, as well as at the booting stage for tissue expression analysis of *Tn1a*. At the no‐tillering stage, *Tn1a* was abundantly expressed in the leaves. The highest expression was observed in the roots during the tillering stage. Compared with the tiller development period, higher expression levels of *Tn1a* were found at the stem base and tiller during the booting stage (Figure 4a). Meanwhile, the *Tn1a* expression level gradually increased in the low‐tiller varieties (LT) after entering the tiller development stage, whereas the high‐tiller varieties (HT) consistently maintained a lower level of expression (Figure S11).

The amino acid alignment revealed a high degree of sequence homology between Tn1a and several C2‐domain‐containing proteins (Figure S12a). Phylogenetic analysis indicated that it belongs to a subfamily of single C2‐domain proteins in plants (Figure S12b). Moreover, the transmembrane region of Tn1a spans amino acids 6 to 29 (Figure S13). To confirm the expression pattern of *Tn1a*, *ProTn1a::GUS* transgenic plants were generated. The results indicated strong GUS activity in the tiller buds and roots (Figure 4b–j). The 35S:*Tn1a*‐GFP vector was transformed into rice protoplasts and tobacco leaves to validate the subcellular localisation of Tn1a. The results showed that Tn1a was localised to the plasma membrane, and non‐synonymous variants in the coding region did not affect its protein localisation (Figure 4k). Collectively, these results indicate that *Tn1a* encodes a membrane‐localised C2‐domain protein.

### Tn1a interacts with OsCCC1 and affects intracellular K^+^ content

**Figure 5:**
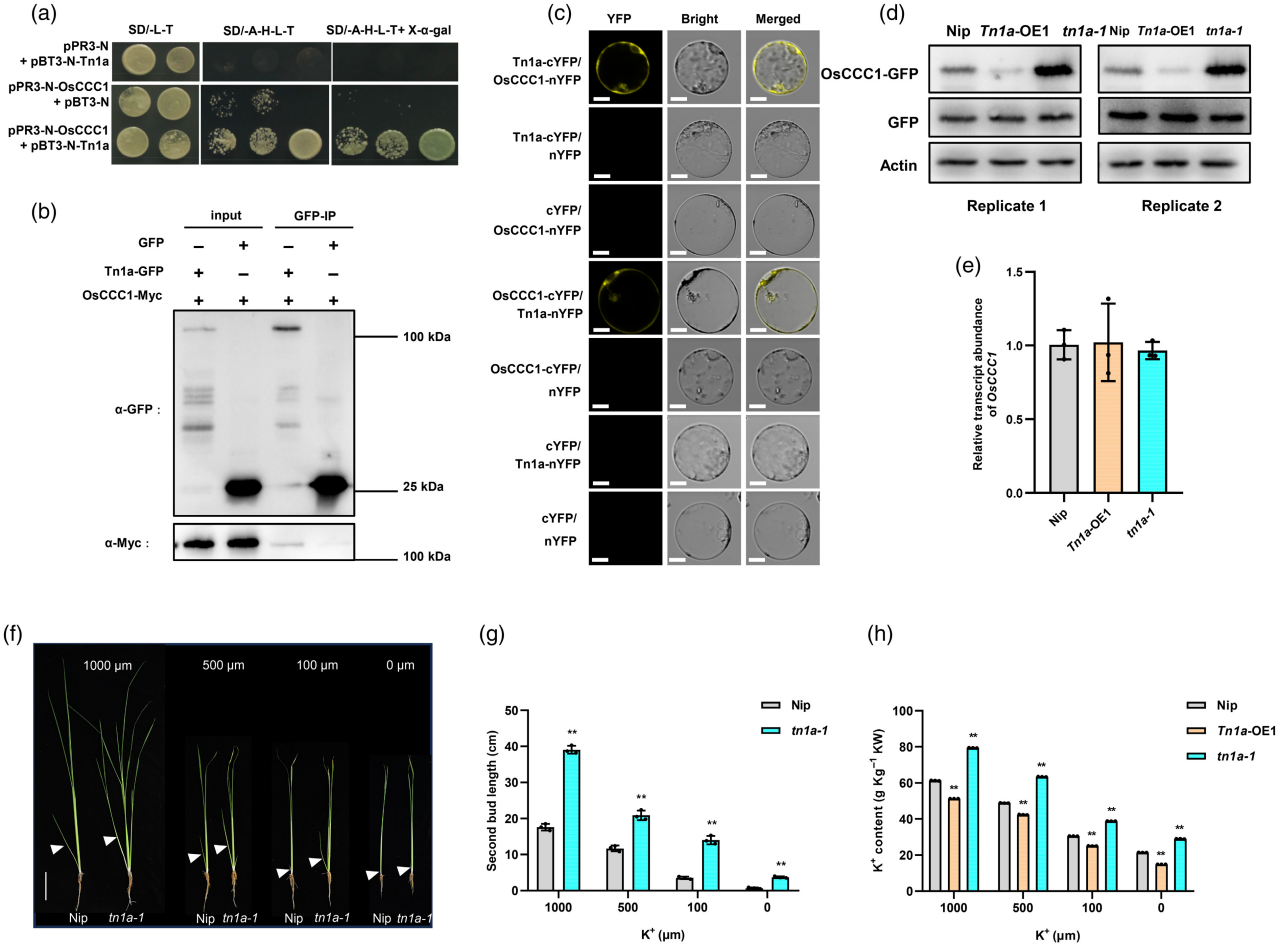
Tn1a Interacts with OsCCC1 at the plasma membrane and alters intracellular K^+^ content. (a) Tn1a interacts with OsCCC1 in yeast. Yeast cells were grown on synthetic defined (SD) medium lacking Leu and Trp (‐L‐T), SD medium lacking Ade, His, Leu and Trp (‐A‐H‐L‐T), SD medium lacking Ade, His, Leu and Trp (‐A‐H‐L‐T) with X‐β‐gal, respectively. (b) Co‐IP assay shows the interaction between Tn1a and OsCCC1 in rice protoplasts. (c) BiFC assay shows the interaction between Tn1a and OsCCC1 in rice protoplasts. The YFP signal was visualised using a confocal microscope. Scale bar = 20 μm. (d) OsCCC1 protein abundance in Nip, *Tn1a*‐OE1 and *tn1a‐1* protoplasts. GFP control and OsCCC1‐GFP were co‐transferred into protoplasts to ensure transformation efficiency. OsCCC1 was detected by an anti‐GFP antibody. Actin served as a control. (e) Expression levels of *OsCCC1* in Nip, *Tn1a*‐OE1 and *tn1a‐1* protoplasts after transformation. Data are presented as the mean ± SD (*n* = 3). (f) Tiller bud appearance of Nip and *tn1a‐1* plants under different K^+^ concentrations. Here, 7‐day‐old rice seedlings were grown for 6 weeks in a hydroponic medium containing K^+^ as the concentrations indicated. The white triangle indicates the second tiller buds. Scale bar = 10 cm. (g) Second bud length of Nip and *tn1a‐1* plants shown in (f). (h) K^+^ content in the shoots of Nip, *Tn1a*‐OE1 and *tn1a‐1* plants under different K^+^ concentrations. Values in (g, h) are mean ± SD (*n* = 3). *P*‐values were determined using a two‐sided Student's *t*‐test (**P < 0.01).

To explore the potential mechanisms of *Tn1a* regulation of tillering, we screened a yeast two‐hybrid (Y2H) library using Tn1a as bait and identified OsCCC1 (cation‐chloride cotransporter 1) as an interacting partner of Tn1a in yeast (Figure 5a). To further confirm this interaction in rice, a co‐immunoprecipitation (Co‐IP) assay was performed on rice protoplasts co‐transformed with Tn1a‐GFP and OsCCC1‐Myc constructs, which successfully demonstrated the interaction between Tn1a‐GFP and OsCCC1‐Myc (Figure 5b). In addition, a bimolecular fluorescence complementation (BiFC) assay was also performed on rice protoplasts. The reconstitution of yellow fluorescent protein (YFP) was observed at the plasma membrane when co‐transformed with Tn1a‐cYFP and OsCCC1‐nYFP, or Tn1a‐nYFP and OsCCC1‐cYFP constructs, and no yellow fluorescence was observed in the other controls (Figure 5c). We investigated whether Tn1a affects OsCCC1 protein content in plants. In rice protoplasts, with consistent *OsCCC1* expression levels, the OsCCC1 protein content in *tn1a‐1* was significantly higher than that in Nip, whereas it was significantly lower in *Tn1a*‐OE1 (Figure 5d,e). These results suggest that Tn1a interacts with OsCCC1 both in vitro and in vivo and might influence the OsCCC1 protein levels.

Given that OsCCC1 is a K^+^ transporter protein and regulates K^+^ transport, the second tiller bud length and plant growth of Nip and *tn1a‐1* under 1000, 500, 100 and 0 μm K^+^ conditions were examined. The results showed that K^+^‐deficiency inhibited the tiller bud length in both the Nip and *tn1a‐1* mutants, and had a stronger inhibitory effect on Nip (Figure 5f,g). In addition, shoot length, root length, and shoot fresh weight were higher in Nip than in *tn1a‐1* under normal K^+^ conditions, and both Nip and *tn1a‐1* showed some degree of decrease in these traits as the K^+^ concentration was reduced, but the decrease in *tn1a‐1* was smaller than that in Nip (Figure S14). In addition, at different concentrations of K^+^, the *Tn1a*‐OE1 line had a lower K^+^ content than Nip, whereas the knockout line *tn1a‐1* was higher than the wild type (Figure 5h). Given that TCP21 and Tb2 can directly repress the transcriptional level of *Tn1a*, we examined K^+^ content in *TCP21* and *Tb2* transgenic lines, and the results showed that there were more K^+^ in *TCP21*‐OE and *Tb2*‐OE lines and less K^+^ in *TCP21‐*C lines compared to Nip (Figure S15). Thus, *TCP21* and *Tb2* positively regulate plant K^+^ content. Together, these results support the notion that Tn1a alters intracellular K^+^ content and inhibits tiller bud length by interacting with OsCCC1.

### The regulation of tillering by *Tn1a* involves in a wide range of pathways

**Figure 6:**
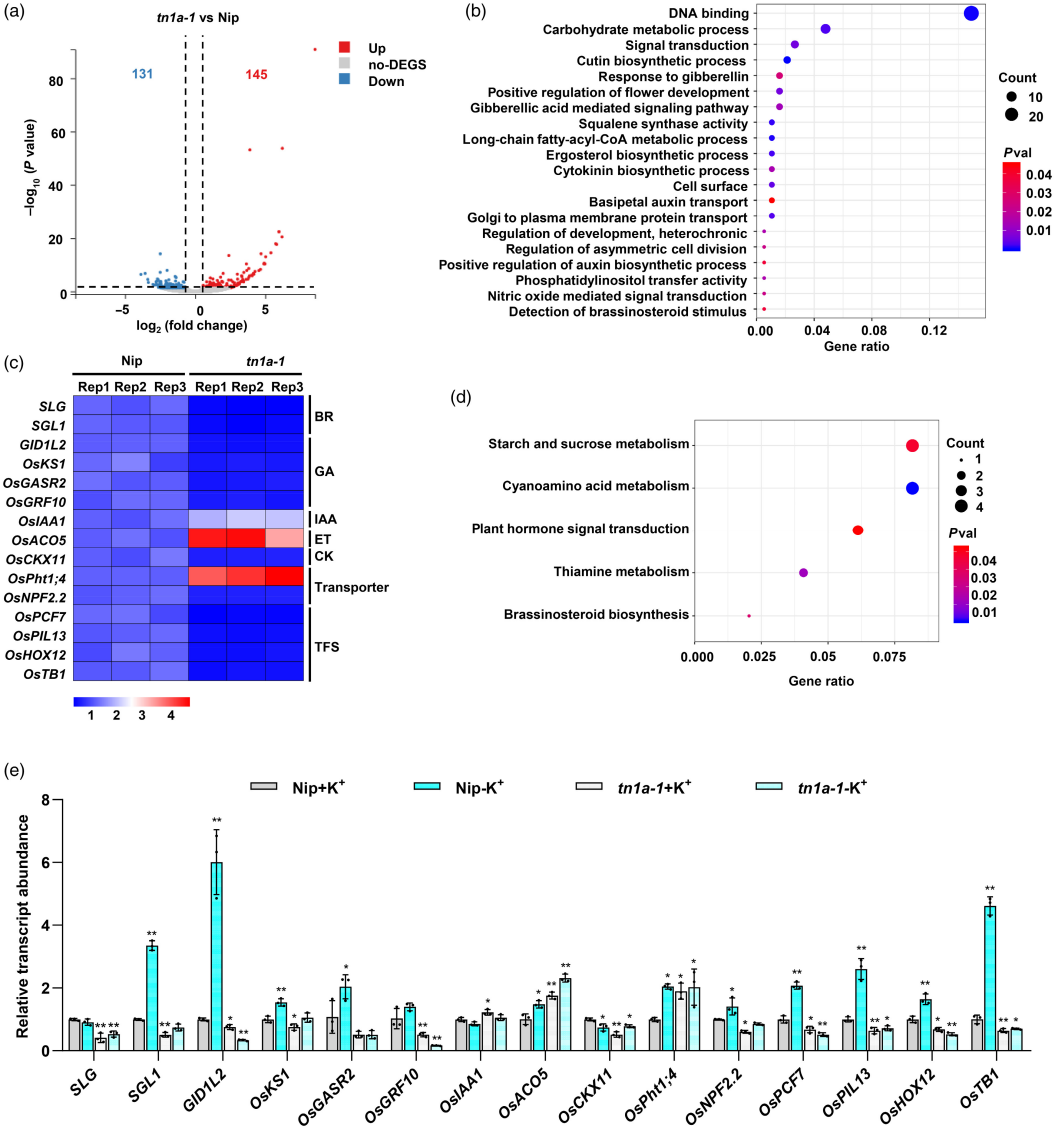
*Tn1a* reprograms global gene expression. (a) Volcano plot of differentially expressed genes (DEGs) between Nip and *Tn1a‐1*. (b) Gene ontology analysis showing the top 20 biological pathways enriched in DEGs. *P* values were adjusted using the Benjamini–Hochberg correction. (c) Heatmap representation of *SLG*, *SGL1*, *GID1L2*, *OsKS1*, *OsGASR2*, *OsGRF10*, *OsIAA1*, *OsACO5*, *OsCKX11*, *OsPht1;4*, *OsNPF2.2*, *OsPCF7*, *OsHOX12*, *OsTB1* expression at the stem bases of Nip and *Tn1a‐1*, as determined by RT‐qPCR. (d) Kyoto Encyclopedia of Genes and Genomes (KEGG) analysis showing the top 5 biological pathways enriched in DEGs. *P* values were adjusted by Benjamini–Hochberg correction and only the significant categories (*P* < 0.05) are displayed. (e) Relative stem base abundances of mRNAs transcribed from the above genes in Nip and *tn1a‐1* under normal K^+^ (1000 μmol) or K^+^‐deficient (0 μmol) conditions. Data are presented as the mean ± SD (*n* = 3). Statistical significance was determined using a two‐sided *t*‐test (*P < 0.05, **P < 0.01).

To further explore the regulation pathway of *Tn1a*, RNA sequencing (RNA‐seq) analysis was performed using shoot bases from the Nip and *tn1a‐1* mutants. Using the threshold of *P* < 0.05 and the value of fold change >2 relative to Nip, 276 differentially expressed genes (DEGs) were obtained, which included 145 up‐regulated and 131 down‐regulated genes in the *tn1a‐1* mutant (Figure 6a). Gene Ontology (GO) term analysis revealed that these DEGs were enriched in biological processes involved in DNA binding, signal transduction, gibberellic acid‐mediated signalling pathways, cytokinin biosynthetic processes, detection of brassinosteroid stimuli and nitric oxide mediated signal transduction. The Kyoto Encyclopedia of Genes and Genomes (KEGG) analysis also revealed that processes such as starch and sucrose metabolism, plant hormone signal transduction, and brassinosteroid biosynthesis were significantly enriched (Figure 6b,d). Because phytohormones, elemental transporter proteins and transcription factors play important roles in the regulation of rice tillering, the relative transcript abundances of 15 associated genes were compared using qPCR. Among these genes, the expression of *OsIAA1*, *OsACO5*, and *OsPht1;4* was increased in the *tn1a‐1* mutant, whereas the expression of the remaining genes decreased (Figure 6c). The expression profile of *Tn1a* was then checked in response to different phytohormones in roots and shoots using RiceXPro Version 3.0 (Sato *et al*., 2013), it can be observed that the expression of *Tn1a* in roots was induced by GA, CK, and ABA but was inhibited by IAA, while the expression of *Tn1a* was induced by CK and ABA in shoots (Figures S16 and S17). Collectively, these results suggest that *Tn1a* for tillering regulation may be achieved by affecting the expression levels of genes related to phytohormones, transporter proteins, and transcription factors.

Finally, to test whether the effect of *Tn1a* on intracellular K^+^ content affected downstream gene expression, the expression levels of these genes were examined at the stem base of wild‐type and *tn1a‐1* mutants under normal and K^+^‐deficient conditions. Under the K^+^‐deficient conditions, the expression levels of *SGL1*, *GID1L2*, *OsKS1*, *OsNPF2.2*, *OsPCF7*, *OsPIL13*, *OsHOX12* and *OsTB1* were significantly increased in Nip compared to the normal condition; however, there was no significant change in *tn1a‐1* (Figure 6e). These results suggest that *Tn1a* is involved in the regulation of these genes by K^+^.

### *Tn1a* enhances the yield and adaptation to low potassium in rice during domestication and breeding

**Figure 7:**
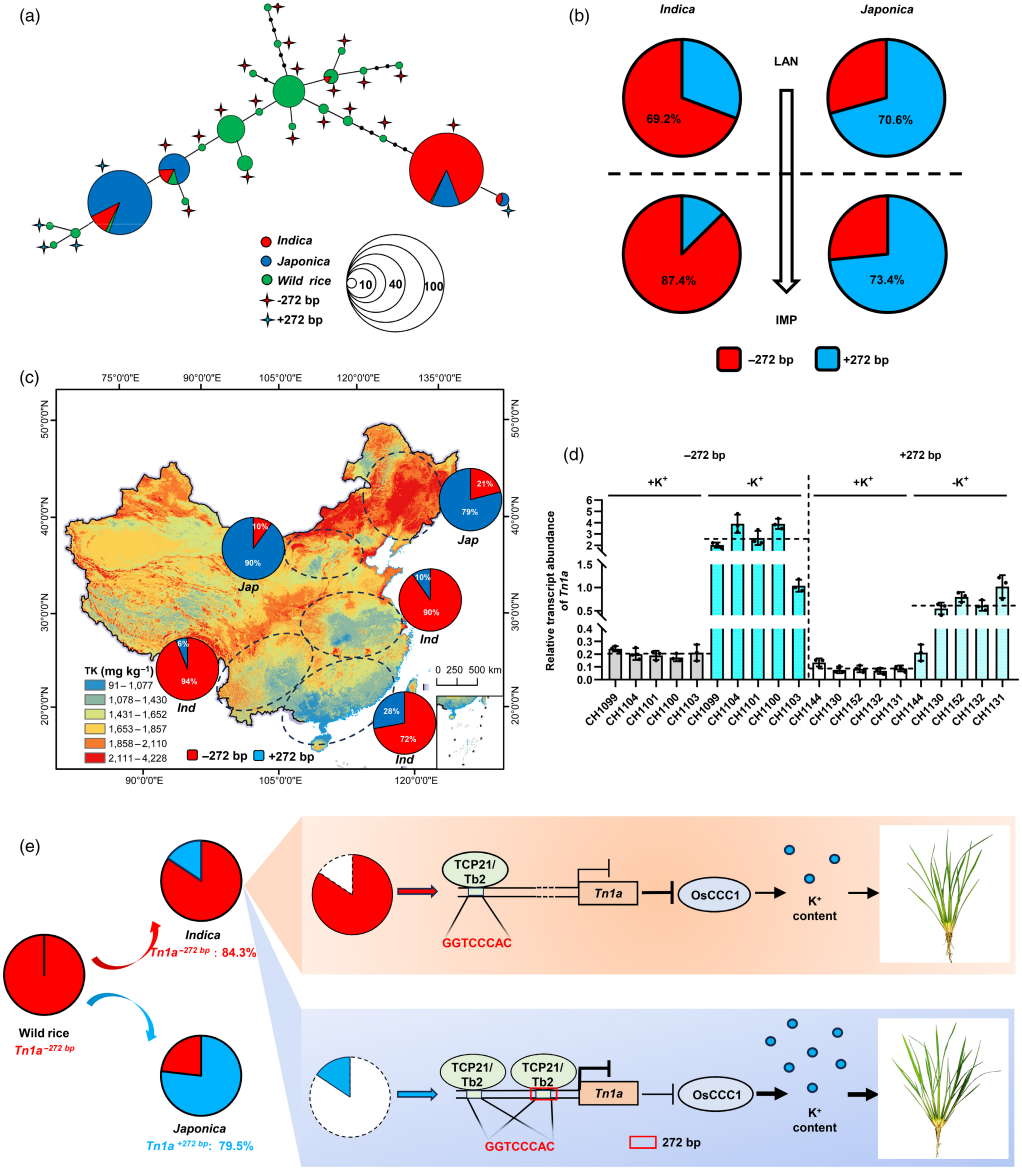
Evolutionary relationship and geographical adaptation of the 272 bp insertion/deletion. (a) Minimum spanning tree for the *Tn1a* region based on 287 cultivated and 71 diverse wild rice sequences. (b) Allele frequency analysis of 272 bp insertion/deletion during rice breeding. Pie charts above and below the horizontal line represent landraces (LAN) and improved varieties (IMP). (c) 272 bp insertion/deletion significantly correlated with soil K^+^ content. Geographic distribution of soil K^+^ content and *Tn1a* allele frequency in different rice subgroups in China. (d) *Tn1a* expression in *indica* subpopulation grouped by the 272 bp indel under normal K^+^ (1000 μmol) or K^+^ deficient (0 μmol) conditions. Data are mean ± SD (*n* = 3). (e) A proposed working model demonstrates the role of 272 bp insertion/deletion in *Tn1a* for the regulation of tillering in rice. 272 bp insertion enhances TCP21/Tb2 binding and transcriptional repression activity and resulted in attenuated repression of OsCCC1 protein content, thereby promoting an increase in plant K^+^ content and tiller number.

To analyse the evolutionary relationship of the 272 bp insertion/deletion in cultivated and wild rice, a minimum‐spanning tree was constructed using a combination of 71 wild rice and 287 cultivated rice accessions (Figure 7a). The results showed that the 272 bp deletion originates in wild rice, and was mainly retained in the *indica* subpopulation. The 272 bp insertion in the *japonica* subpopulation was probably caused by gene mutations during the later domestication and improvement processes. To explore whether the 272 bp indel was subjected to selection during *Tn1a* domestication or breeding, the nucleotide diversity and Tajima's *D* values of wild rice, *O. sativa*, *ssp. indica* and *ssp. japonica* was determined. Nucleotide diversity was significantly lower in *O. sativa* (π = 0.0002171) than in the wild rice (π = 0.0025501). In addition, the significantly positive Tajima's *D* value in *O. sativa* indicated that *Tn1a* was subjected to balanced selection. Furthermore, a 272 bp deletion in *ssp. indica* and a 272 bp insertion in *ssp*. *japonica* were subjected to positive selection (Table S5).

To understand the breeding utilisation of *Tn1a*, changes in allelic frequencies were investigated during the breeding of 339 cultivated rice accessions (Table S4). The results showed that the 272 bp deletion and 272 bp insertion were mainly present in *indica* and *japonica* subpopulations, respectively, regardless of the landraces or improved variety. Moreover, the proportion of the 272 bp deletion in the *indica* subpopulation increased significantly during breeding and improvement (Figure 7b). Because *Tn1a* regulates rice development by altering intracellular K^+^ content and *japonica* subpopulations are planted mainly in the northern China and *Indica* subpopulations are planted mainly in the south, we speculated that the allele distribution of *Tn1a* in the two rice subgroups attributes to their environmental adaptation, especially the soil potassium content in different geographical locations. We collected data on soil total potassium (TK) content across the country, projected all varieties back to their origin locations and compared the relationship between the *Tn1a* allele frequency and soil K^+^ content in different regions (Figure 7c). In the *indica* subpopulation, the proportion of *Tn1a* ^*+272 bp*^ allele in rice varieties was significantly higher in South China (28%, 12/43) than in East (10%, 3/31) and Southwest China (6%, 2/33), presenting negative correlation with the soil K^+^ content, which was significantly lower in South China than in the other two regions. Similar results were obtained for the *japonica* subpopulation, with lower soil potassium levels, but a higher proportion of *Tn1a* ^*+272 bp*^ allele in central China (90%, 9/10) than in northeastern China (79%, 15/19). These findings indicated that the *Tn1a* ^*+272 bp*^ allele may have contributed to the geographical adaptation of rice varieties to potassium‐poor soils (Figure 7c). Then, we compared the tiller numbers of Nip and the knockout mutant *tn1a‐1* in different K^+^ concentrations. The result showed that *tn1a‐1* had significantly more tillers than the wild type under both +K^+^ and −K^+^ conditions (Figure S18). Next, under normal and K^+^ deficient conditions, we examined the expression levels of *Tn1a*, *TCP21* and *Tb2* in *indica* varieties with or without the 272 bp insertion respectively. K^+^‐deficiency (−K^+^) induced the expression of *Tn1a* in both genotypes, and the *Tn1a* expression abundance of varieties containing the *Tn1a* ^*+272 bp*^ allele was lower than those containing the *Tn1a* ^−*272 bp*^ allele, regardless of the condition (Figure 7d). In contrast, the effect of K^+^ on the expression levels of *TCP21* and *Tb2* in both types of varieties was opposite to that of *Tn1a* (Figure S19). But, compared to the *Tn1a* ^−*272 bp*^ allele, the response of the *Tn1a* ^*+272 bp*^ allele to K^+^‐deficiency was significantly weakened (*P* = 0.0048) and thus contributed to the varieties with this allele higher K^+^ utilisation efficiency. Collectively, the *Tn1a* ^*+272 bp*^ might have been preserved in rice varieties in potassium‐poor regions during domestication; however, its proportion decreased in cultivated rice during breeding utilisation.

Given that the role of 272 bp indel in the *Tn1a* promoter is associated with geographical adaptation in rice, a molecular marker for this functional locus was designed. The results showed that varieties with 272 bp fragments had more tillers and higher K^+^ content than varieties without it (Figure S20). Therefore, the 272 bp indel could be used in marker‐assisted selection breeding for high yield and K^+^ efficiency.

## Discussion

The tiller number is an important factor in constituting the ideal plant architecture and is crucial for grain yield (Jiao *et al*., 2010). Thus, it is essential for crop improvement to elucidate the genetic and molecular bases of tiller formation in rice and mine favourable allelic variations in natural populations. In this study, we cloned *Tn1a*, which has a negative regulatory effect on rice tiller number, through genome‐wide association analysis, focusing on the dynamic changes in tiller number. We proposed a model for the role of *Tn1a* in the regulation of tiller number and its divergence during rice domestication (Figure 7e).

In this study, we found that *Tn1a* CRISPR knockout lines had significantly lower plant height and shorter root system but increased tiller number, secondary branch number, grain number per panicle and grain yield per plant (Figure 1d,f; Figure S4), indicating that the pleiotropic effects of *Tn1a* may be attributed to the higher nutrient uptake efficiency and K^+^ content in *Tn1a* knockout lines compared to Nip. In contrast, the lower K^+^ content may lead to yellower leaves in the overexpression lines (Figure 1e). *Tn1a* encodes a membrane‐localised C2 domain‐containing protein (OsC2DP). This protein is involved in plant growth and development, stress responses, and hormone responses in Arabidopsis and rice (Fu *et al*., 2019; Vaddepalli *et al*., 2014; Zhang *et al*., 2022). Here, we report that Tn1a interacts with OsCCC1, and increases the protein content of OsCCC1 in *tn1a‐1* mutants (Figure 5). However, it remains unclear how Tn1a affects the protein content of OsCCC1 and whether it affects OsCCC1 protein activity. Identification of the interaction between Tn1a and OsCCC1 will help clarify the underlying regulatory mechanisms. Given that OsCCC1 is involved in K^+^ transport, we found that K^+^ content was significantly elevated in *tn1a‐1* mutants and may have modulated the uptake of other nutrients as well as hormonal signals promoting tiller bud elongation (Figures 5e,f, 6). This suggests a potential downstream role for phytohormones and nutrients in the regulation of tillering by *Tn1a*. Numerous studies have demonstrated an important link between phytohormones and nutrients in the regulation of rice tillering. Nitrogen promotes the accumulation of DNR1 protein levels, leading to a decrease in auxin, which promotes tillering (Zhang *et al*., 2021). In addition, *REGULATOR OF N‐RESPONSIVE RSA ON CHROMOSOME 10* (*RNR10*) encodes a F‐box protein that mono‐ubiquitinates DNR1 and inhibits its degradation, thereby antagonising auxin accumulation (Huang *et al*., 2023). Nodulation Signalling Pathway 1 (NSP1) and NSP2 respond to Pi deficiency and directly regulate the expression of SL synthesis genes, thereby regulating tillering (Yuan *et al*., 2023). These studies provide evidence of the complex interactions between plant hormones and nutrients. In this study, elevated intracellular K^+^ levels and altered expression of phytohormone‐related genes were observed in the *tn1a‐1* mutants. In addition, *Tn1a* was induced by GA, CK and ABA, whereas it was inhibited by IAA, indicating that phytohormones and K^+^ content play important roles in the involvement of *Tn1a* in the regulation of tillering in rice (Figures S16 and S17). However, the mechanism through which *Tn1a* regulates tillering requires further investigation in future studies.

The genotype of *Tn1a* ^−*272 bp*^ derived from wild rice is mainly retained in the *indica* subpopulation, and the genotype *Tn1a* ^*+272 bp*^ arose as a result of a mutation in the *japonica* subpopulation during later domestication and improvement processes. To date, the TCP transcription factor family has been reported to be extensively involved in the regulation of tillering in rice, but different genes have different roles in tillering; for example, OsTB1 and OsTCP19 negatively regulate tillering, whereas TCP21 and Tb2 positively regulate (Liu *et al*., 2021; Lyu *et al*., 2020; Takeda *et al*., 2003; Wang *et al*., 2021). Our study found that TCP21 and Tb2 could bind to the TCP recognition site in the 272 bp segment (Figure 3). The two promoter types are associated with the differential regulation of *Tn1a* expression due to differences in TCP21 and Tb2 binding activities. The 272 bp insertion sequence contains a TCP recognition site that results in enhanced repression of the promoter by TCP21 and Tb2, leading to relatively weak expression of *Tn1a* and a greater number of tillers. In contrast, the absence of this repression at the *Tn1a* ^−*272 bp*^ promoter resulted in a relatively robust expression of *Tn1a* and a lower tiller number. However, it remains unclear whether the transcriptional repression of *Tn1a* by TCP21 and Tb2 affects tillering regulation by *Tn1a*.

The soil environment is important for the adaptation of plants to the local natural environment (Guerrero *et al*., 2018). Nonetheless, how plants produce such adaptations remains largely unknown. Here, we report that allelic variation in *Tn1a* promotes geographic adaptation of rice to soil potassium. Our study also suggests a scheme for rice adaption to different K^+^ levels in the soil. During rice domestication in different geographic locations in China, the K^+^ content of the soil may vary considerably and is significantly higher in the north than in the south of the rice‐growing region (Figure 7c). In potassium‐deficient regions, especially in *indica* rice‐planting areas of China, *Tn1a* ^*+272 bp*^ is retained more in K^+^ deficient environments, whereas in regions with relatively high potassium content, the relaxation of selection pressure leads to a lower proportion of *Tn1a* ^*+272 bp*^. Excess applications of fertilisers, including potash, is often carried out to meet food demands against human population growth, which may result in negative selection for *Tn1a* ^*+272 bp*^ in modern cultivars. Our results showed that *Tn1a* ^−*272 bp*^ was subjected to positive selection during the domestication of *indica* rice, as well as its utilisation in breeding (Figure 7b; Table S5). Therefore, to achieve high yields with low fertiliser application rates, it is necessary to reintroduce the lost alleles into modern cultivars. We designed and validated molecular markers based on variant loci to facilitate selective breeding in the future. Of course, as to the low frequency in *indica* and modern cultivars, we may consider and validate one interesting and possible explanation in future that the *Tn1a* ^*+272 bp*^ allele may have some disadvantageous effect on *indica* rice.

## Materials and methods

### Plant materials and growth conditions

In the current study, wild‐type rice (*Oryza sativa* L.) Nipponbare (Nip), along with its CRISPR mutants, *Tn1a*‐overexpressing lines, RNAi lines and three complementation lines, were used. All plants were grown under natural paddy conditions at the Shangzhuang Experimental Station of China Agricultural University in Beijing, or at Sanya, in Hainan Province.

### Vector generation and genetic transformation

To construct the CRISPR/Cas9 vector, a 19 bp PAM sequence from the *Tn1a* CDS was selected for specific recognition and cloned into the SK‐gRNA vector, as previously described (Wang *et al*., 2015). To construct the p*35S*:*Tn1a* overexpression (OE) vector, a 2025 bp *Tn1a* cDNA without the stop codon was inserted into the *SpeI* and *KpnI* sites of the binary plant expression vector pMDC1307. Moreover, a 308 bp fragment of *Tn1a* cDNA was inserted downstream of the Ubi promoter in pTCK303 to construct a *Tn1a* RNA interference (RNAi) vector. To construct the *ProTn1a::GUS* vector, a genomic fragment of the *Tn1a* promoter region starting 2856 bp upstream of the ATG initiation codon was amplified and cloned into the *PmeI* and *AscI* sites of the binary plant expression vector pMDC162. The 2.6 kb promoter fragment upstream of *Tn1a* in TSN and *Tn1a* cDNA in Nip were fused to pMDC163 to construct the genomic DNA complementation constructs *ProTn1a* ^*TSN*^::*Tn1a* ^*Nip*^, *ProTn1a* ^*Nip*^::*Tn1a* ^*TSN*^ and *ProTn1a* ^*TSN*^ *::Tn1a* ^*TSN*^. All three models were constructed similarly. These constructs were transformed into Nipponbare (Nip) by the *Agrobacterium*‐mediated transformation method to generate transgenic plants. Homozygous T_2_ generation transgenic lines were verified using hygromycin selection and PCR analysis.

### RNA extraction and RT‐qPCR

Total RNA was extracted from different plant tissues using the TRIzol reagent (Invitrogen, Carlsbad, CA, USA) and reverse‐transcribed into cDNA using M‐MLV reverse transcriptase (Takara). The expression levels of the target genes were detected using the SYBR Green reagent (Takara) in an Applied Biosystems 7500 Fast Real‐Time PCR System. *OsActin1* served as an internal reference. All experiments were performed with at least three biological replicates.

### Haplotype, nucleotide diversity and selection analysis

We used a worldwide set of 287 cultivated rice accessions selected from the 3 K‐Rice Project (Rice Functional Genomics and Breeding Database, RFGB) (Wang *et al*., 2018b) and 71 wild rice accessions provided by Dr. Song Ge (Table S3). The 272 bp insertion/deletion alleles of *Tn1a* in the rice accessions were identified from the PCR products and then subjected to genotyping. Variation sites, including seven single nucleotide polymorphisms (SNPs), which were detected at three stages simultaneously in the promoter and coding regions, could also cause non‐synonymous mutations in the coding sequence. One insertion/deletion polymorphism was used for haplotype analysis. For nucleotide diversity, the *Tn1a* genomic sequence was used for analysis, and their π values and Tajima's *D* were calculated and performed using DNASP 5.10 (Librado and Rozas, 2009).

### Phylogenetic and minimum spanning tree analyses

The SNPs and indels used in the haplotype analysis were also used for the phylogenetic tree analysis. A neighbour‐joining tree was constructed using MEGA 5.0 (Tamura *et al*., 2011) and then visualised and annotated using EvolView (Zhang *et al*., 2012). The common variation sites of cultivated and wild rice were used for minimum spanning tree analysis, and the minimum spanning tree was generated following a previously described procedure (Yu *et al*., 2018).

### Alignment and phylogenetic analysis

Sequence alignment of the C2‐domain‐containing proteins was performed using ClustalW. Phylogenetic analysis of the C2‐domain‐containing protein family based on amino acid sequences was performed using the neighbour‐joining method in MEGA version 7.0. Neighbour‐joining analysis was performed using the ‘complete deletion’ option. Support for each node was tested by bootstrap analysis, with 1000 replicates for neighbour‐joining, using a random input order for each replicate.

### Transcriptional activity assay

The CDSs of Tb2 and TCP21 in Nip were amplified and inserted into the pGreenII 62‐SK vector and used as effectors the 2‐Kb promoter fragments of the four haplotypes (Hap1‐4) in the *indica* subpopulation and the TSN, Nip, Nip plus 272 bp fragments were amplified and inserted into the pGreenII 0800‐LUC vector and used as reporters. Reporters containing the four haplotype promoters were transferred to rice protoplasts to compare their promoter activity. Similarly, a reporter containing the TSN, Nip, and Nip plus 272 bp promoters was transferred into rice protoplasts to verify the effect of the 272 bp insertion on *Tn1a* promoter activity.

Effectors containing Tb2 and TCP21 CDSs and reporters containing TSN, Nip, and Nip plus a 272 bp fragment promoter were co‐transformed into rice protoplasts to assay the effect of Tb2 and TCP21 on 272 bp insertion. 35S: REN was used as an internal control. Firefly luciferase (LUC) and Renilla luciferase (REN) activity was quantified using a dual‐luciferase reporter assay system (Promega, Madison, WI, USA). The LUC/REN ratio was used as a measure of relative promoter activity.

### EMSA

The EMSA was performed as previously described (Shao *et al*., 2019). Full‐length cDNAs of Tb2 and TCP21 were cloned into the pMAL‐C5X vector, and MBP‐Tb2 and MBP‐TCP21 were expressed in *Escherichia coli BL21* (*DE3*) cells and subsequently purified according to the manufacturer's instructions. The recombinant protein was then incubated with the biotin‐labelled probe, unlabeled probe and unlabeled‐mprobe using the EMSA/Gel‐Shift Kit (GS009; Beyotime, China) for 20 min at room temperature. The reaction mixture was separated on a native PAGE gel. The binding signal was detected using BeyoECL Moon A and BeyoECL Moon B (Beyotime).

### GUS‐staining assay

As previously described (Zhang *et al*., 2018), tissues from transgenic plants harbouring the *ProTn1a::GUS* reporter construct were incubated in a GUS staining solution at 37°C for 12 h in the dark.

### Subcellular localisation

*Tn1a* ^*TSN*^ and *Tn1a* ^*Nip*^ cDNA were amplified from TSN and Nip, respectively, and cloned into the pMDC1300‐GFP vector to obtain *Pro35S::Tn1a* ^*TSN*^ *‐GFP* and *Pro35S::Tn1a* ^*Nip*^ *‐GFP* constructs. The resulting vectors and PIP2‐mCherry, a plasma membrane marker, were co‐transfected into rice protoplasts. After culturing for 16 h at 28°C, fluorescence signals were observed as previously described (Zhang *et al*., 2023).

### Protein interaction analysis

The membrane system yeast two‐hybrid assays were performed as described previously (Hu *et al*., 2021). Yeast cells (NYM51) were co‐transformed with pPR3‐N‐OsCCC1 constructs and pBT3‐N‐Tn1a or the empty vector pPR3‐N and pBT3‐N. Yeast cells were grown on ‐Leu/−Trp or ‐His/−Ade/−Leu/−Trp‐selective media for 2–3 days.

The BiFC assay was performed on rice protoplasts. Full‐length of *Tn1a* and *OsCCC1* in Nip were amplified and inserted into the pSPYNE and pSPYCE, respectively. The constructs were co‐transfected into rice protoplasts, and the YFP fluorescence signal was detected using a confocal laser‐scanning microscope (excitation wavelength, 488 nm). *Tn1a‐GFP* and *OsCCC1‐Myc* vectors were co‐transformed into wild‐type rice protoplasts and maintained in the dark for 15 h. After centrifugation, the total protein in the protoplasts was extracted with IP buffer (50 mM Tris–HCl, pH 7.5, 150 mM NaCl, 0.2% NP‐40, 5 mM DTT, and 1 × protease inhibitor cocktail), followed by incubation with GFP beads (Chromo Tek, Germany) for 2 h. After immunoprecipitation, the samples were washed five times with wash buffer for 5 min each (50 mM Tris–HCl, pH 7.5, 150 mM NaCl, and 0.1% NP‐40) each time. Immunoblot analysis was performed using an anti‐MYC antibody (Sigma‐Aldrich, USA).

### Determination of potassium accumulation using ICP‐MS

The content of total potassium in the plant samples was determined by Inductive Coupled Plasma Emission Spectrometer (ICP‐OES). Sample processing and testing were performed as previously described (Yan *et al*., 2019).

### RNA‐seq

mRNA was extracted from the stem base of the axillary buds of Nip and the *tn1a* mutant 55 days after transplanting. Three biological replicates were used for each plant sample. Sample extraction and Illumina sequencing were performed at the Beijing Genome Institute (Wuhan, China). The clean reads were mapped to the rice genome (MSU‐RGAP 7.0). Gene expression levels were calculated using RSEM. A volcano map of the DEGs between Nip and *tn1a* mutants was plotted using Hiplot (https://hiplot.com.cn/). A heat map was constructed using TBtools (https://github.com/CJ‐Chen/TBtools). GO enrichment of the DEGs was performed using PhyPer (http://www.geneontology.org/). The significance of the GO terms was corrected using FDR <0.05.

### Geographical distribution of soil potassium content

This dataset is a nationwide high‐resolution spatial distribution map of the total soil potassium. The horisontal spatial resolution was 90 m, and the depth of the soil layer in the vertical direction was 0–5 cm. This dataset was obtained from the National Earth System Science Data Center of National Science and Technology Infrastructure of China (http://www.geodata.cn). The base map was created using ArcGIS software.

### Primers

Primers used in this study are listed in Table S6.

## Conflict of interest

The authors declare that they have no competing interests.

## Author contributions

T.Y., X.M., H.Z. and Z.Z. designed the research. T.Y. and X.M. performed most of experiments. Q.Z., L.L., R.Z. and Y.W. performed part of the experiments. N.U. modified the paper. T.Y. and X.M. analysed the data. H.Z. and Z.Z. conceived and supervised the project. T.Y. and X.M. wrote the manuscript.

## Supporting information


**Figure S1** Expression analysis of the other nine candidate genes of qTn1.7 in tiller base.
**Figure S2** Global gene expression profile of three candidate genes.
**Figure S3** Identification and phenotype of Tn1a transgenic lines.
**Figure S4** Panicle performance of different transgenic plants of Tn1a.
**Figure S5** Nucleotide diversity analysis for the promoter region and CDS of Tn1a.
**Figure S6** Phylogenetic analysis of Tn1a in a natural rice population.
**Figure S7** Tiller number of different haplotypes and genotypes among different tillering periods.
**Figure S8** Proportional distribution of 272 bp indel in indica and japonica subpopulation.
**Figure S9** DNA sequence polymorphisms of Tn1a in Nip and TSN.
**Figure S10** Characterisation of the axillary bud of different Tn1a transgenic plants.
**Figure S11** The dynamic expression analysis of Tn1a in stem base during the tiller development.
**Figure S12** Sequence alignment and phylogenetic analysis of Tn1a.
**Figure S13** Prediction of the transmembrane regions of Tn1a.
**Figure S14** Tn1a negatively regulates potassium deficiency stress in rice seedings.
**Figure S15** TCP21 and Tb2 positively regulate plant K+ content.
**Figure S16** The expression profile of Tn1a in root from rice seedlings treated with phytohormones.
**Figure S17** The expression profile of Tn1a in shoot from rice seedlings treated with phytohormones.
**Figure S18** Tn1a knockout promotes tiller number in K+ deficient soils.
**Figure S19** TCP21 and Tb2 expression in indica subpopulation grouped by the 272 bp indel under normal K+ (1000 μmol) and K+ deficient (0 μmol) conditions.
**Figure S20** Breeding utilisation of 272 bp indel as a molecular marker.

**Table S1** Summary of candidate genes and their significantly associated SNP at three stages in *qTn1.7*.
**Table S2** Information of low tiller and high tiller varieties for the expression level detection of candidate genes in *qTn1.7*.
**Table S3** Information of cultivated and wild rice used in the phylogenetic tree and minimum spanning tree analyses.
**Table S4** Information of cultivated rice used in the allele frequency analyses.
**Table S5** Nucleotide diversity analysis and neutral test of *Tn1a*.
**Table S6** Primers used in this study.

## Data Availability

The data that support the findings of this study are available on request from the corresponding author. The data are not publicly available due to privacy or ethical restrictions.
