# Supplementary material for: Natural variation in the Tn1a promoter regulates tillering in rice
Source: Plant Biotechnol J. 2024 Aug 27;22(12):3345–60. doi: 10.1111/pbi.14453 (PMC11606419; doi:10.1111/pbi.14453)
Supplement: Supplementary file 1 — Figure S1 Expression analysis of the other nine candidate genes of qTn1.7 in tiller base. Figure S2 Global gene expression profile of three candidate genes. Figure S3 Identification and phenotype of Tn1a transgenic lines. Figure S4 Panicle performance of different transgenic plants of Tn1a. Figure S5 Nucleotide diversity analysis for the promoter region and CDS of Tn1a. Figure S6 Phylogenetic analysis of Tn1a in a natural rice population. Figure S7 Tiller number of different haplotypes and genotypes among different tillering periods. Figure S8 Proportional distribution of 272 bp indel in indica and japonica subpopulation. Figure S9 DNA sequence polymorphisms of Tn1a in Nip and TSN. Figure S10 Characterisation of the axillary bud of different Tn1a transgenic plants. Figure S11 The dynamic expression analysis of Tn1a in stem base during the tiller development. Figure S12 Sequence alignment and phylogenetic analysis of Tn1a. Figure S13 Prediction of the transmembrane regions of Tn1a. Figure S14 Tn1a negatively regulates potassium deficiency stress in rice seedings. Figure S15 TCP21 and Tb2 positively regulate plant K+ content. Figure S16 The expression profile of Tn1a in root from rice seedlings treated with phytohormones. Figure S17 The expression profile of Tn1a in shoot from rice seedlings treated with phytohormones. Figure S18 Tn1a knockout promotes tiller number in K+ deficient soils. Figure S19 TCP21 and Tb2 expression in indica subpopulation grouped by the 272 bp indel under normal K+ (1000 μmol) and K+ deficient (0 μmol) conditions. Figure S20 Breeding utilisation of 272 bp indel as a molecular marker. [file PBI-22-3345-s001.docx]

**Natural variation in the *Tn1a* promoter regulates tillering in rice**

Tao Yang^1,†^, Xiaoqian Ma^1,2,†^, Quan Zhang^1^, Lin Li^1^, Rui Zhu^1^, An Zeng^1,3^, Wanying Liu^1^, Haixia Liu^1^,Yulong Wang^1^, Shichen Han^1^, Najeeb Ullah Khan^1^, Jinjie Li^1,3^, Zichao Li^1,3^, Zhanying Zhang^1,3,^*, Hongliang Zhang^1,3,4,^*

^1^Frontiers Science Center for Molecular Design Breeding, Key Laboratory of Crop Heterosis and Utilization (MOE), Beijing Key Laboratory of Crop Genetic Improvement, College of Agronomy and Biotechnology, China Agricultural University, Beijing 100193, China.

^2^College of Agriculture, Henan University of Science and Technology, Luoyang 471000, China.

^3^Sanya Institute of China Agricultural University, Sanya 572025, China.

^4^Sanya Nanfan Research Institute of Hainan University, Sanya 572025, China.

**Figure S1** Expression analysis of the other 9 candidate genes of *qTn1.7* in tiller base

**Figure S2** Global gene expression profile of 3 candidate genes

**Figure S3** Identification and phenotype of *Tn1a* transgenic lines

**Figure S4** Panicle performance of different transgenic plants of *Tn1a*

**Figure S5** Nucleotide diversity analysis for the promoter region and CDS of *Tn1a*

**Figure S6** Phylogenetic analysis of *Tn1a* in a natural rice population

**Figure S7** Tiller number of different haplotypes and genotypes among different tillering periods

**Figure** **S8** Proportional distribution of 272 bp indel in *indica* and *japonica* subpopulation

**Figure S9** DNA sequence polymorphisms of *Tn1a* in Nip and TSN

**Figure S10** Characterization of the axillary bud of different *Tn1a* transgenic plants

**Figure S11** The dynamic expression analysis of *Tn1a* in stem base during the tiller development

**Figure S12** Sequence alignment and phylogenetic analysis of *Tn1a*

**Figure S13** Prediction of the transmembrane regions of Tn1a

**Figure S14** *Tn1a* negatively regulates potassium deficiency stress in rice seedings

**Figure S15** *TCP21* and *Tb2* positively regulate plant K^+^ content

**Figure S16** The expression profile of *Tn1a* in root from rice seedlings treated with phytohormones

**Figure S17** The expression profile of *Tn1a* in shoot from rice seedlings treated with phytohormones

**Figure S18** *Tn1a* knockout promotes tiller number in K^+^ deficient soils

**Figure S19** *TCP21* and *Tb2* expression in *indica* subpopulation grouped by the 272 bp indel under normal K^+^ (1000 μmol) and K^+^ deficient (0 μmol) conditions

**Figure S20** Breeding utilization of 272 bp indel as a molecular marker


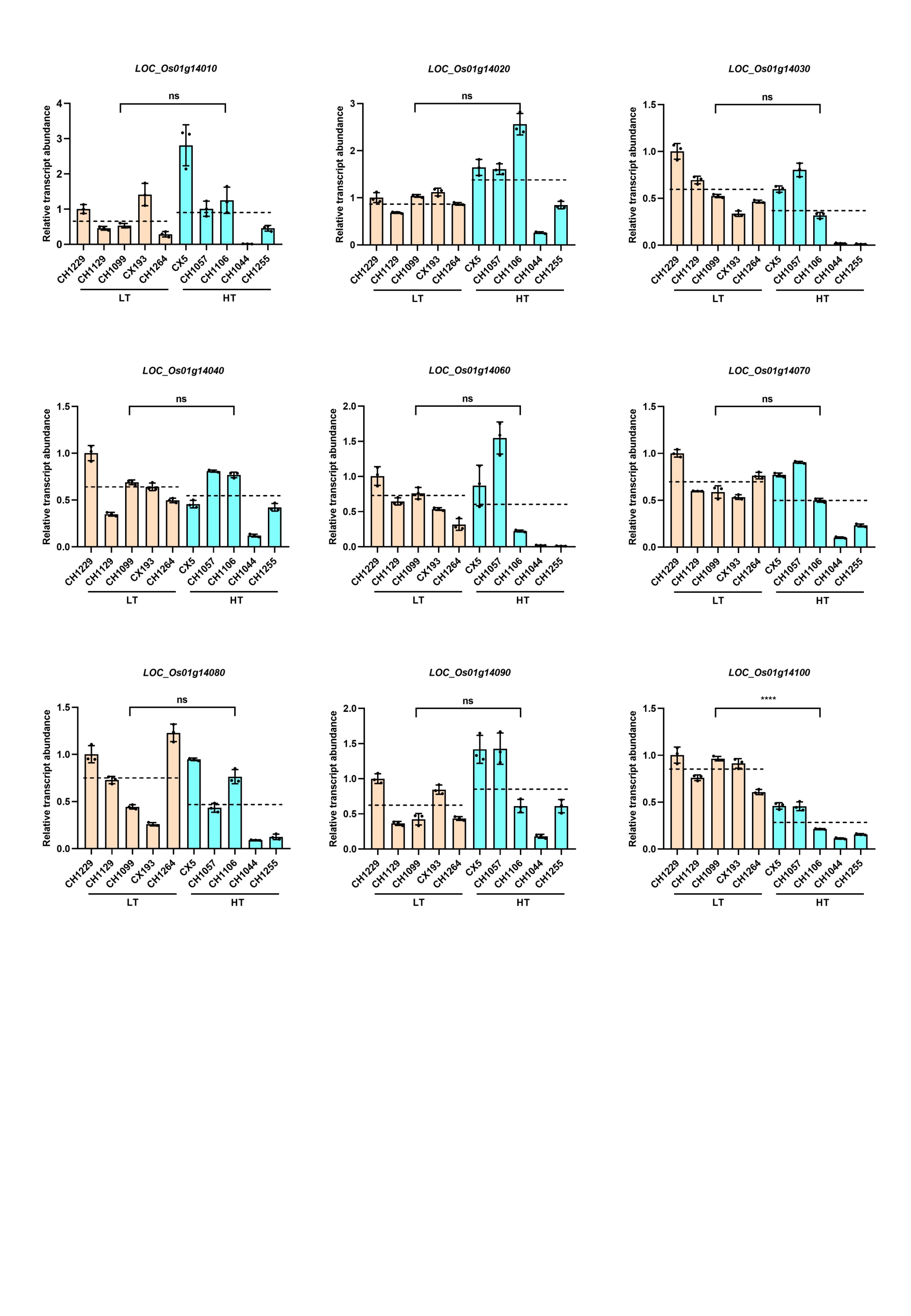


**Figure S1 Expression analysis of the other 9 candidate genes of *qTn1.7* in tiller base**

Relative expression level was calibrated to *OsActin1* gene expression. Data represent mean ± SD (n = 3). Statistical significance was determined by a two-sided *t*-test: not significant (ns), *P*>0.05, *****p* < 0.0001.


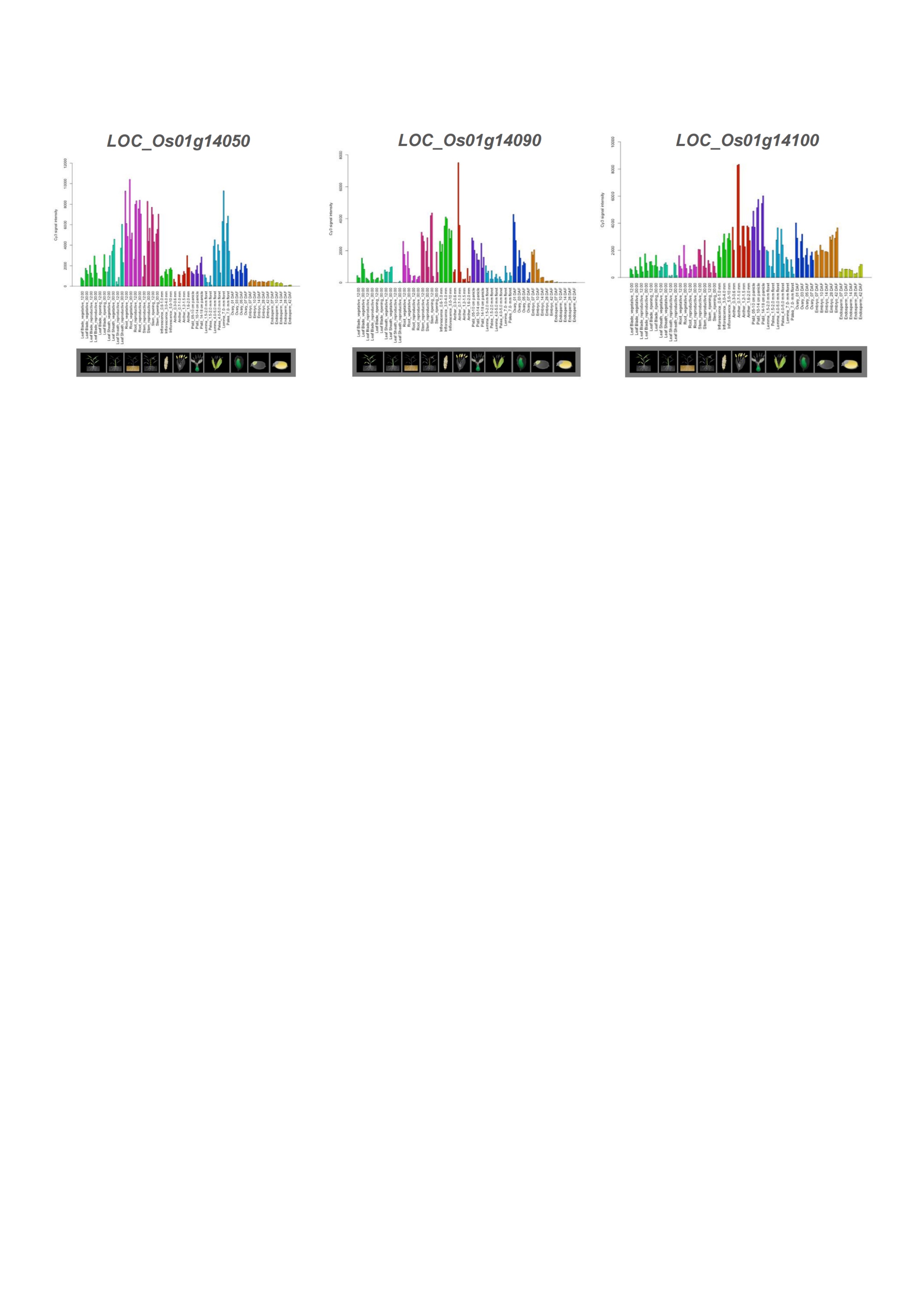


**Figure S2 Global gene expression profile of 3 candidate genes**

The expression data were obtained from the RiceXPro website (http://ricexpro.dna.affrc.go.jp/GGEP/).


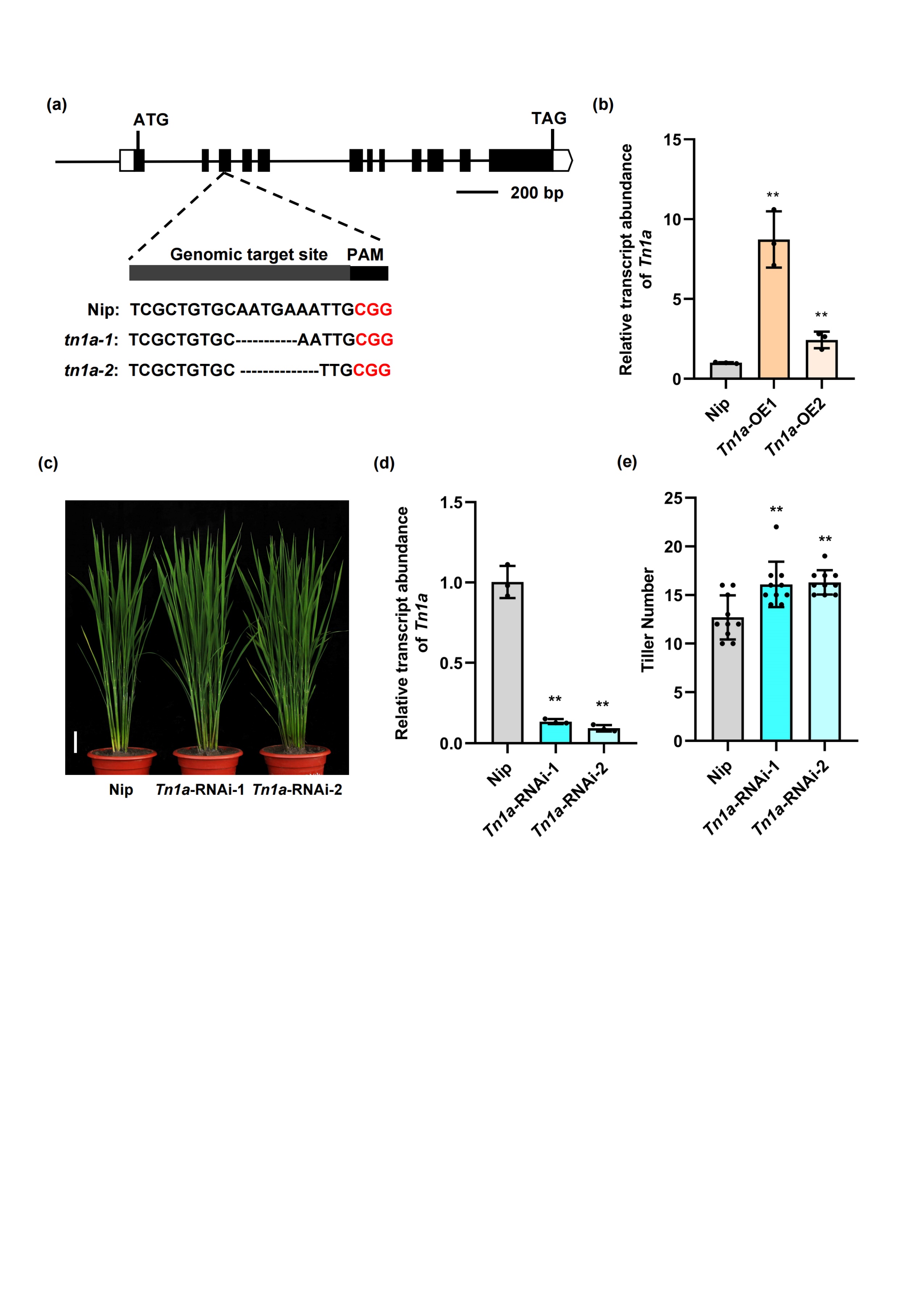


**Figure S3 Identification and phenotype of *Tn1a* transgenic lines**

(a) Schematic diagram of knockout mutants generated using CRISPR/Cas9. Target site in the second exon is indicated by black slash in the *Tn1a* gene model, the target sequence is in gray box, and protospacer-adjacent motif (PAM) sequence is indicated by black box. Black dashes represent deletions. (b) Expression analysis of *Tn1a* in OE lines. Data presented as mean ± SD (n = 3). (c) Plant architecture of Nip, *Tn1a*-RNAi-1 and *Tn1a*-RNAi-2 lines at the tillering stage. Scale bar = 10 cm. (d) Expression analysis of *Tn1a* in RNAi lines. Data represent mean ± SD (n = 3). (e) Tiller numbers of Nip, *Tn1a*-RNAi-1 and *Tn1a*-RNAi-2 lines. Data are presented as mean ± SD (n = 10). statistical significance in (b, d, e) was determined using a two-sided *t*-test (***p* < 0.01).


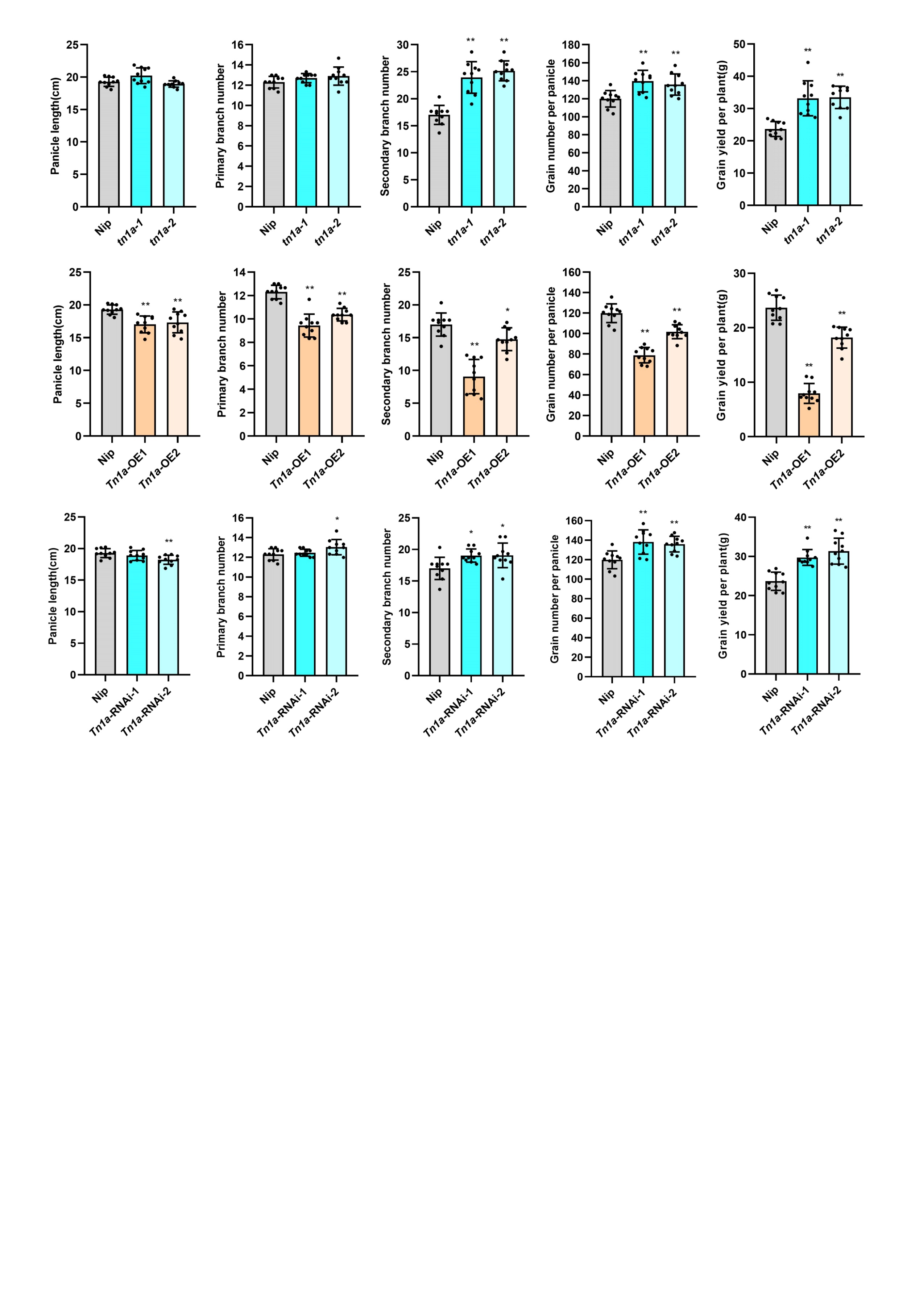


**Figure S4 Panicle performance of different transgenic plants of *Tn1a***

Comparisons of panicle length, primary branch number, secondary branch number, grain number per-panicle and grain yield per plant between Nip and knockout, overexpression, and RNAi lines of *Tn1a*. Data are presented as mean ± SD (n = 10). Statistical significance was determined using a two- sided *t*-test (**p* < 0.05, ***p* < 0.01).


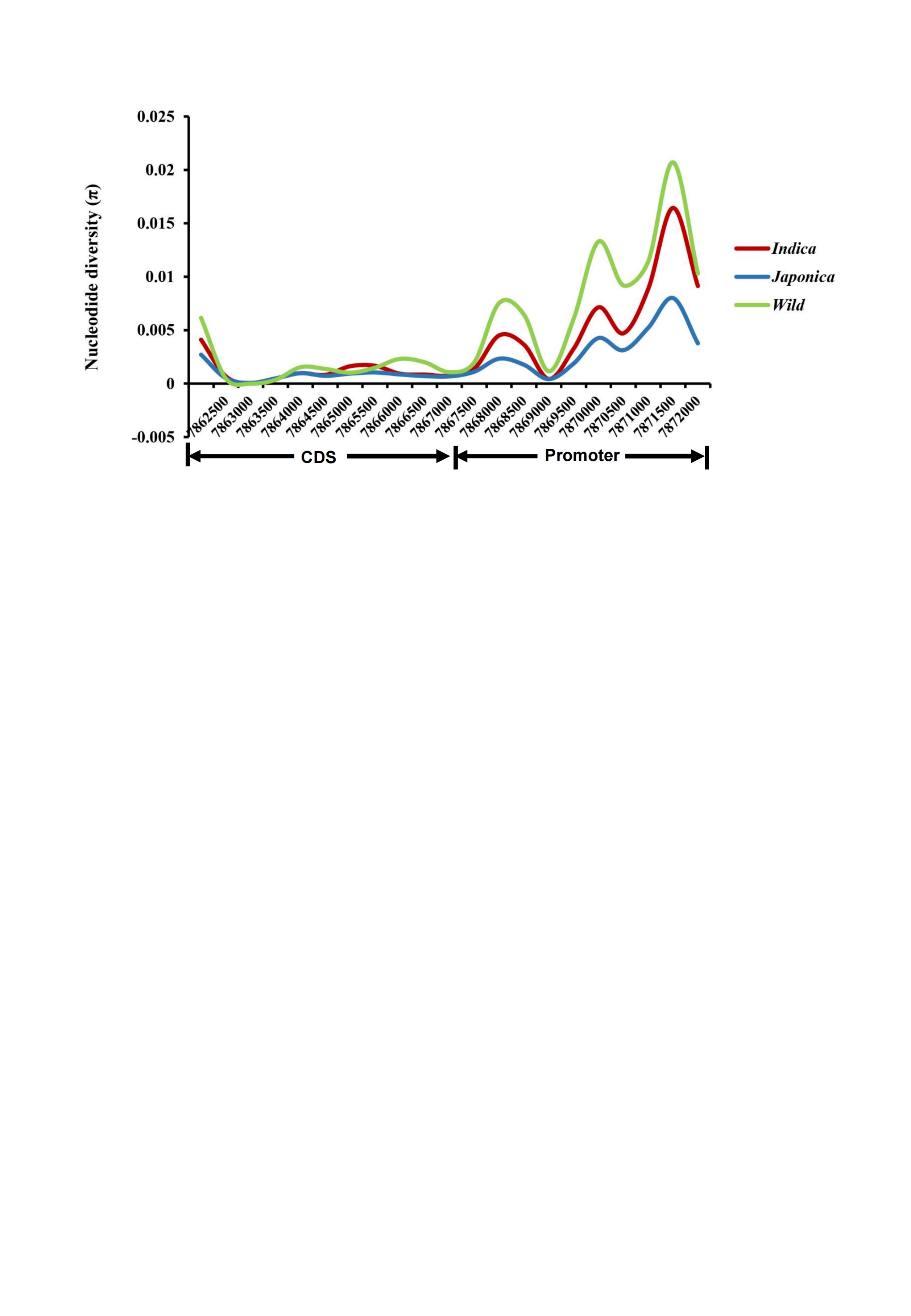


**Figure S5 Nucleotide diversity analysis for the promoter region and CDS of *Tn1a***


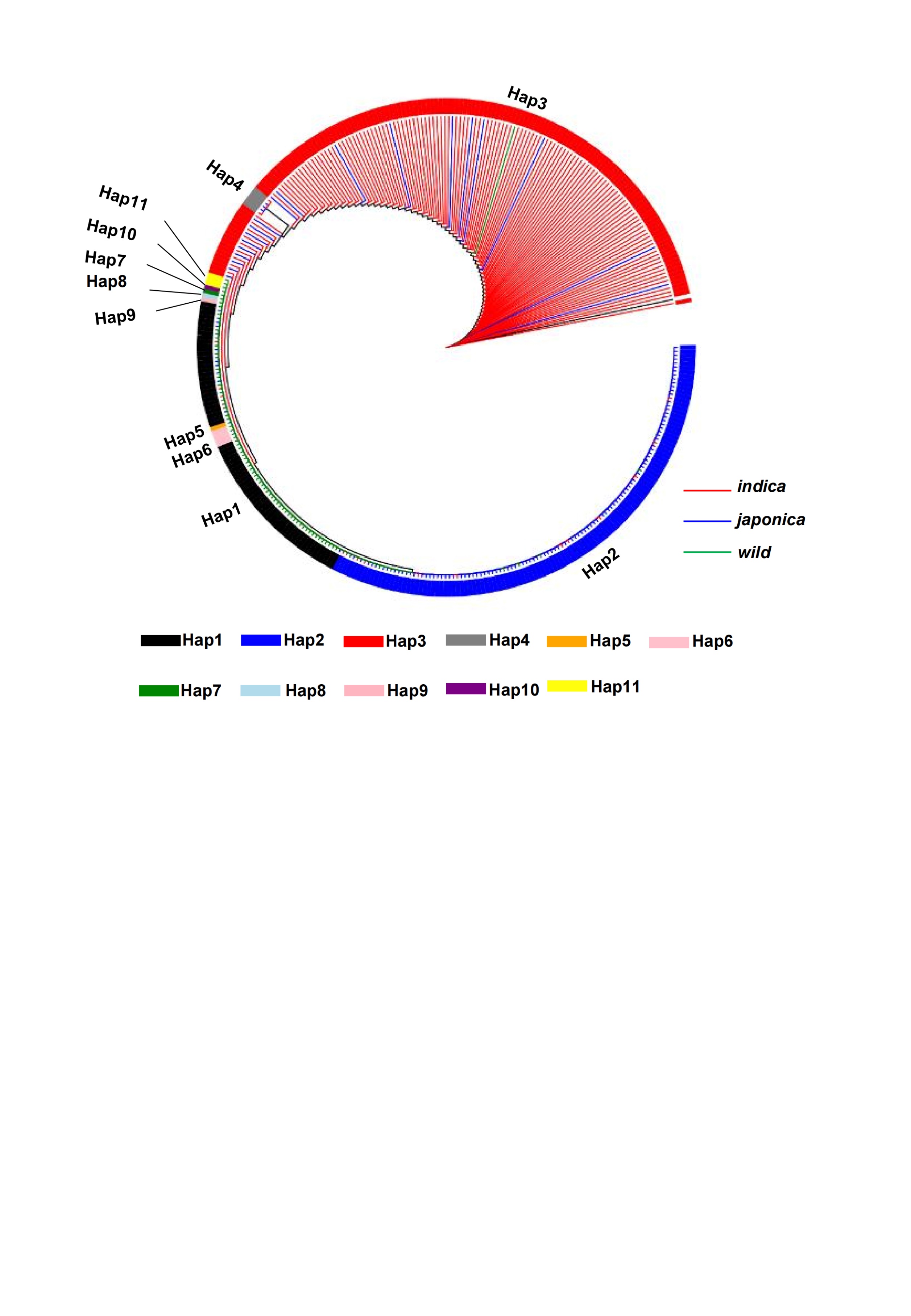


**Figure S6 Phylogenetic analysis of *Tn1a* in a natural rice population**

Phylogenetic tree of *Tn1a* sequence was constructed using the neighbor-joining method with MEGA software (version 7). Color of the outer circle refers to 11 Haplotype; Inner solid colors indicate three ecological groups, including *indica*, *japonica* and *wild*.


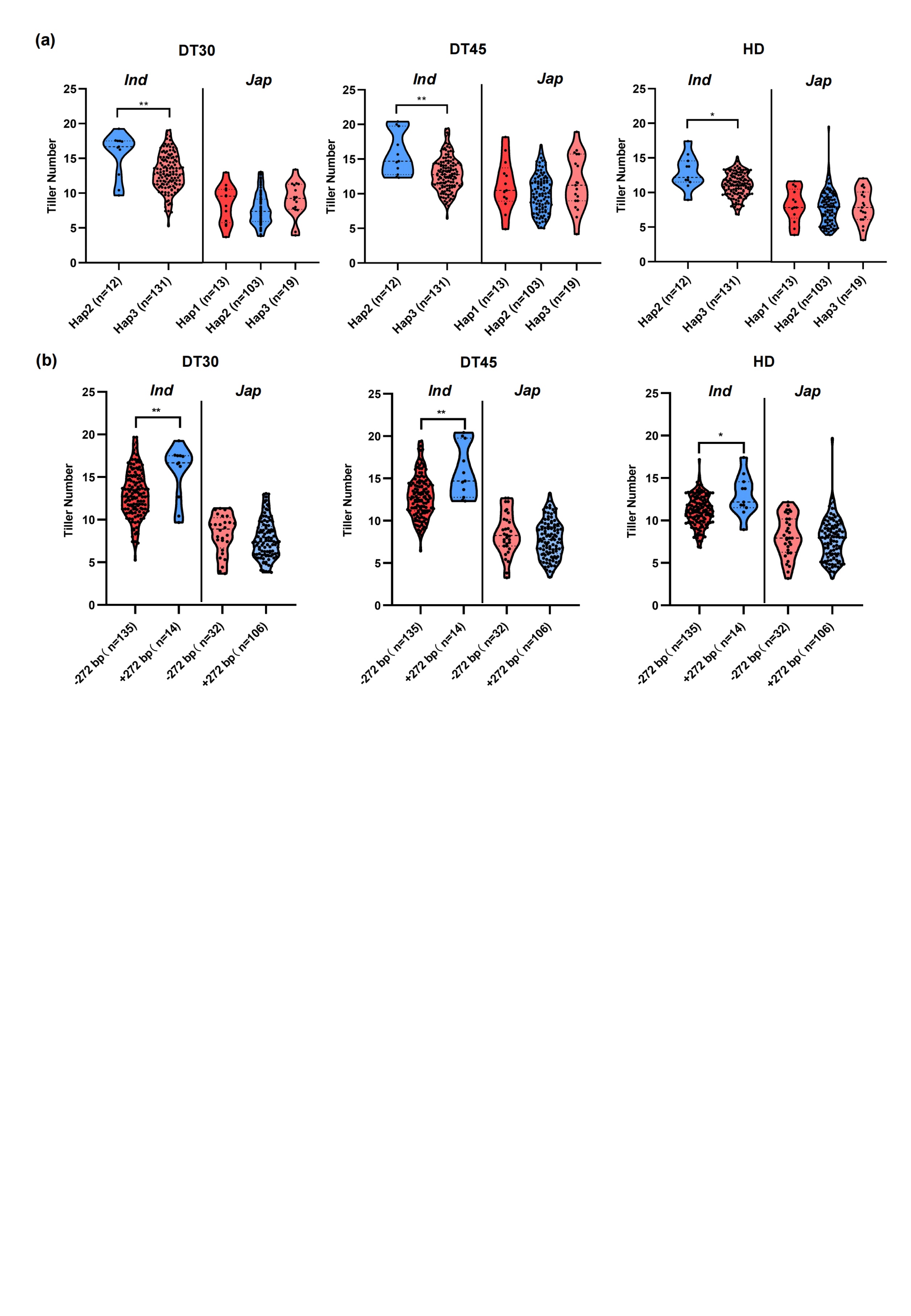


**Figure S7 Tiller number of different haplotypes and genotypes among different tillering periods**

(a) Comparison of tiller number among cultivars grouped by Hap1, Hap2 and Hap3 at DT30, DT45 and HD stage. (b) Distribution of tiller number at DT30, DT45 and HD stage with plants grouped by the 272 bp indel. Statistical significance was determined using a two- sided *t*-test (**p* < 0.05, ***p* < 0.01).


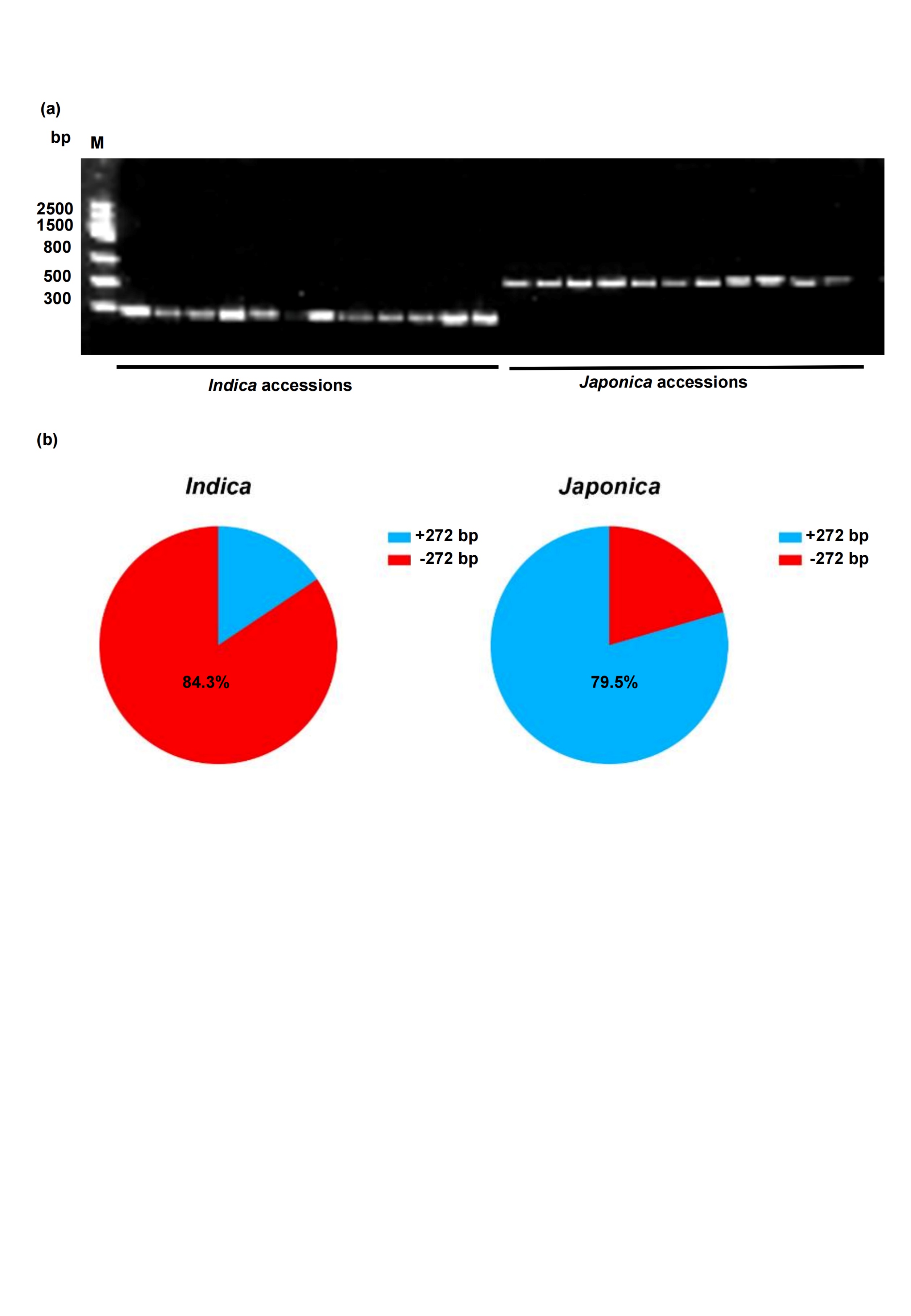


**Figure S8 Proportional distribution of 272 bp indel in *indica* and *japonica* subpopulation**

(a) Agarose gel electrophoresis of PCR amplified products using a primer pair flanking the 272 bp insertion/deletion segment in our collection of 12 *indica* and 11 *japonica* varieties. (b) Proportional distribution of 272 bp insertion/deletion in *indica* and *japonica* varieties in our collection.


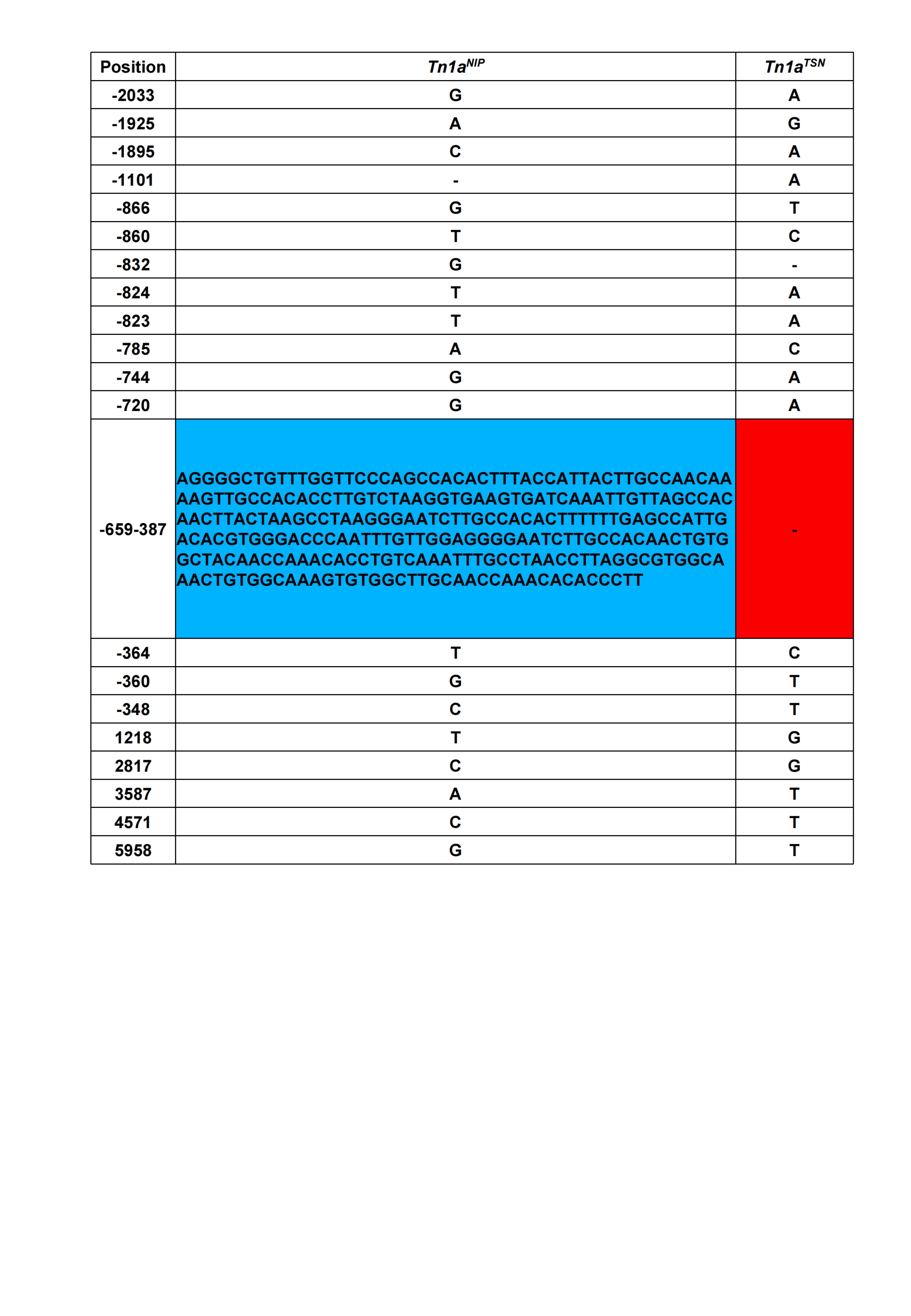


**Figure S9 DNA sequence polymorphisms of *Tn1a* in Nip and TSN**

Complete list of *Tn1a* allelic variations and their positions relative to the*Tn1a* start codon, blue and red boxes indicate Nip and TSN predicted functional variant sites.


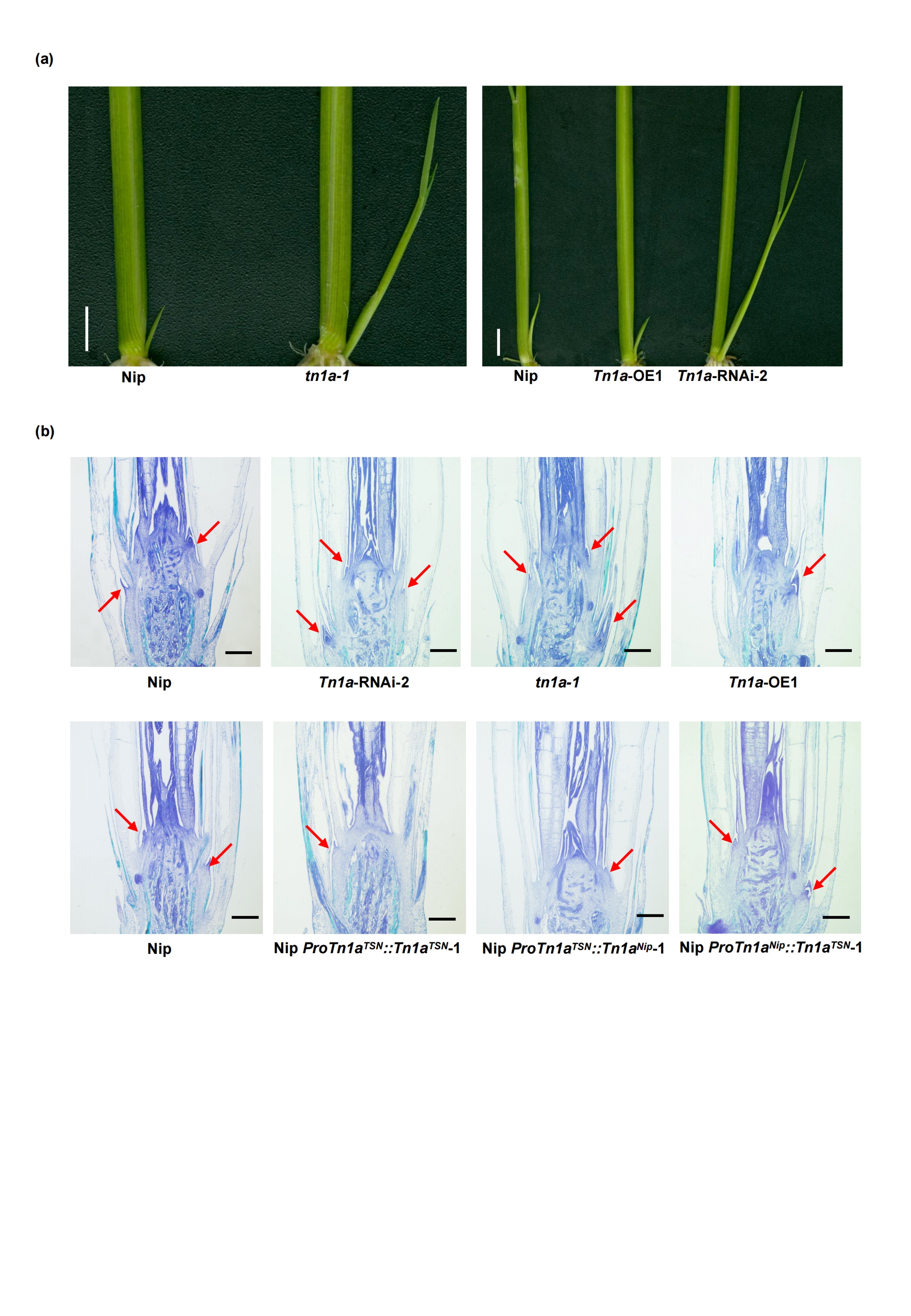


**Figure S10 Characterization of the axillary bud of different *Tn1a* transgenic plants**

(a) Tiller buds in Nip, *tn1a-1*, *Tn1a*-OE1, and *Tn1a-*RNAi-2 plants at 28 days after germination. Tiller bud growth was faster for *tn1a-1* and *Tn1a-*RNAi-2 plants, and slower for *Tn1a*-OE1 than for Nip. Scale bar = 1 cm. (b) Longitudinal sections of shoot apical meristems (SAMs) and axillary buds of Nip, *Tn1a-*RNAi-2, *tn1a-1,* *Tn1a*-OE1, Nip *ProTn1a^TSN^::Tn1a^TSN^*-1, Nip *ProTn1a^TSN^::Tn1a^Nip^*-1, and Nip *ProTn1a^Nip^::Tn1a^TSN^*-1 plants. The positions of axillary buds in these plants are indicated by red arrows. Bars = 100 µm.


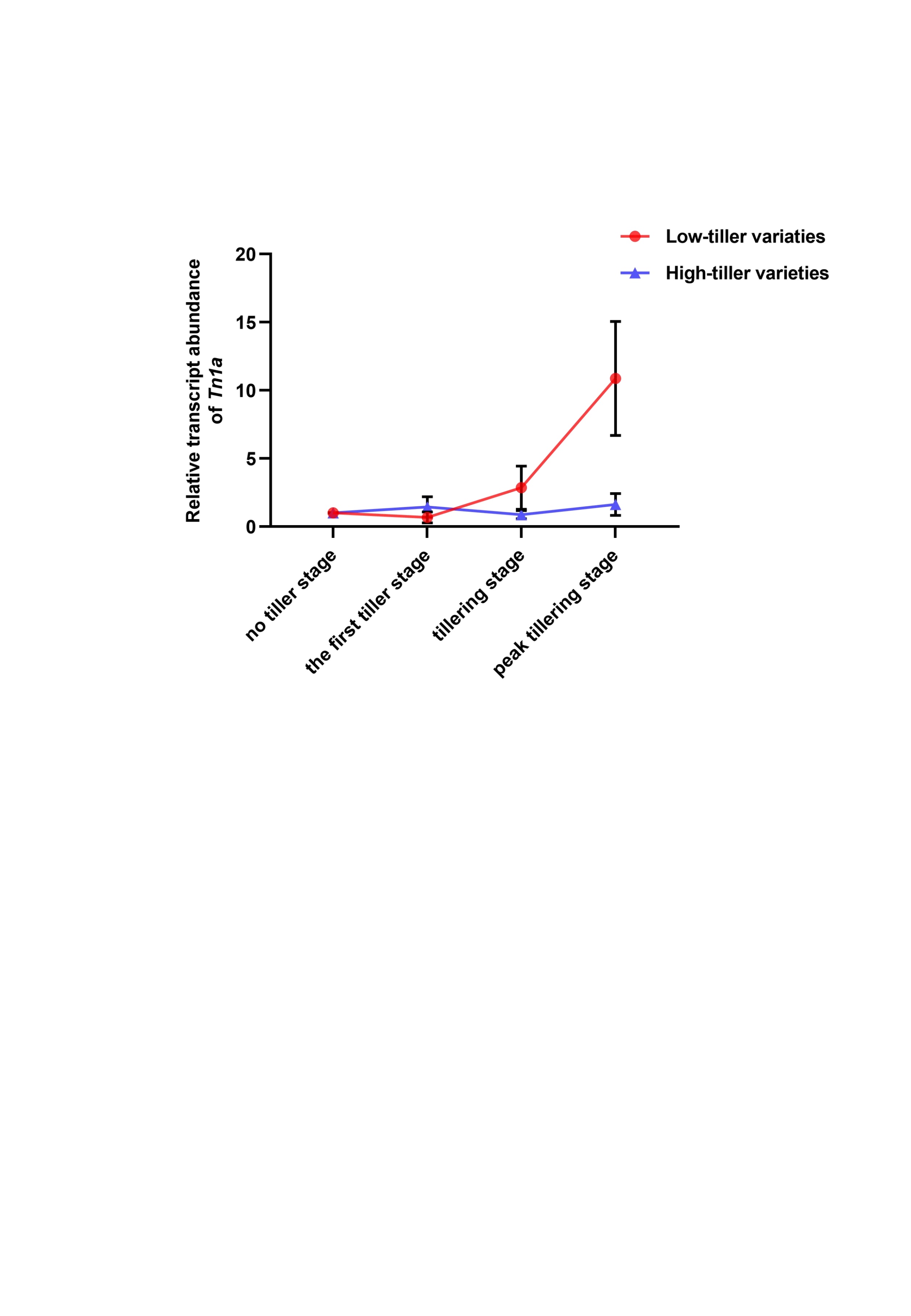


**Figure S11 The dynamic expression analysis of *Tn1a* in stem base during the tiller development**

All varieties were planted in 15 days apart. When the first batch of planted varieties produced the first tiller, samples were taken from the second batch varieties, which was regarded as the no-tillering stage.  The tillering and peak of the tillering stage were calculated according to the dynamic change of the first batch and sampled at the approximate stage of the second batch.


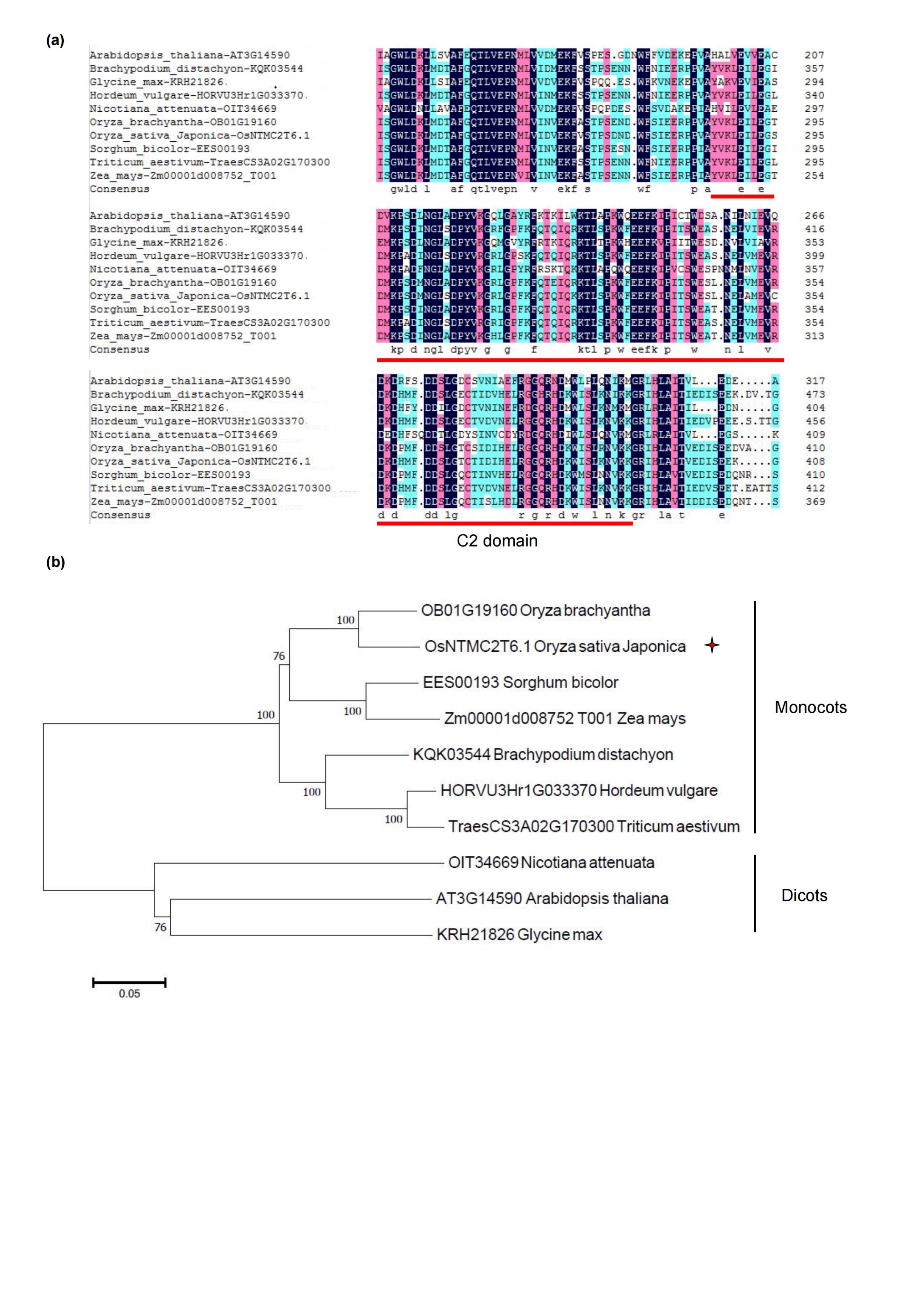


**Figure S12 Sequence alignment and phylogenetic analysis of *Tn1a***

(a) Alignment of amino acid sequences of Tn1a in different plant species. Amino acid residues identical to the consensus C2-domain sequences are shown above the red line. (b) Phylogenetic analysis of Tn1a homologues in rice and other plants. The phylogenetic tree was constructed by the MEGA 6 software based on amino acid sequences. Red star represents Tn1a. OB01G19160, Oryza brachyantha; EES00193, Sorghum bicolor; Zm00001d008752, Zea mays; KQK03544, Brachypodium distachyon; HORVU2Hr1G033370, Hordeum vulgare; TraesCS3A02G170300, Triticum aestivum; OIT34669, Nicotiana attenuata; AT3G14590, Arabidopsis thaliana; KRH21826, Glycine max. Sequence were obtained from EnsemblPlants (http://plants.ensembl.org).


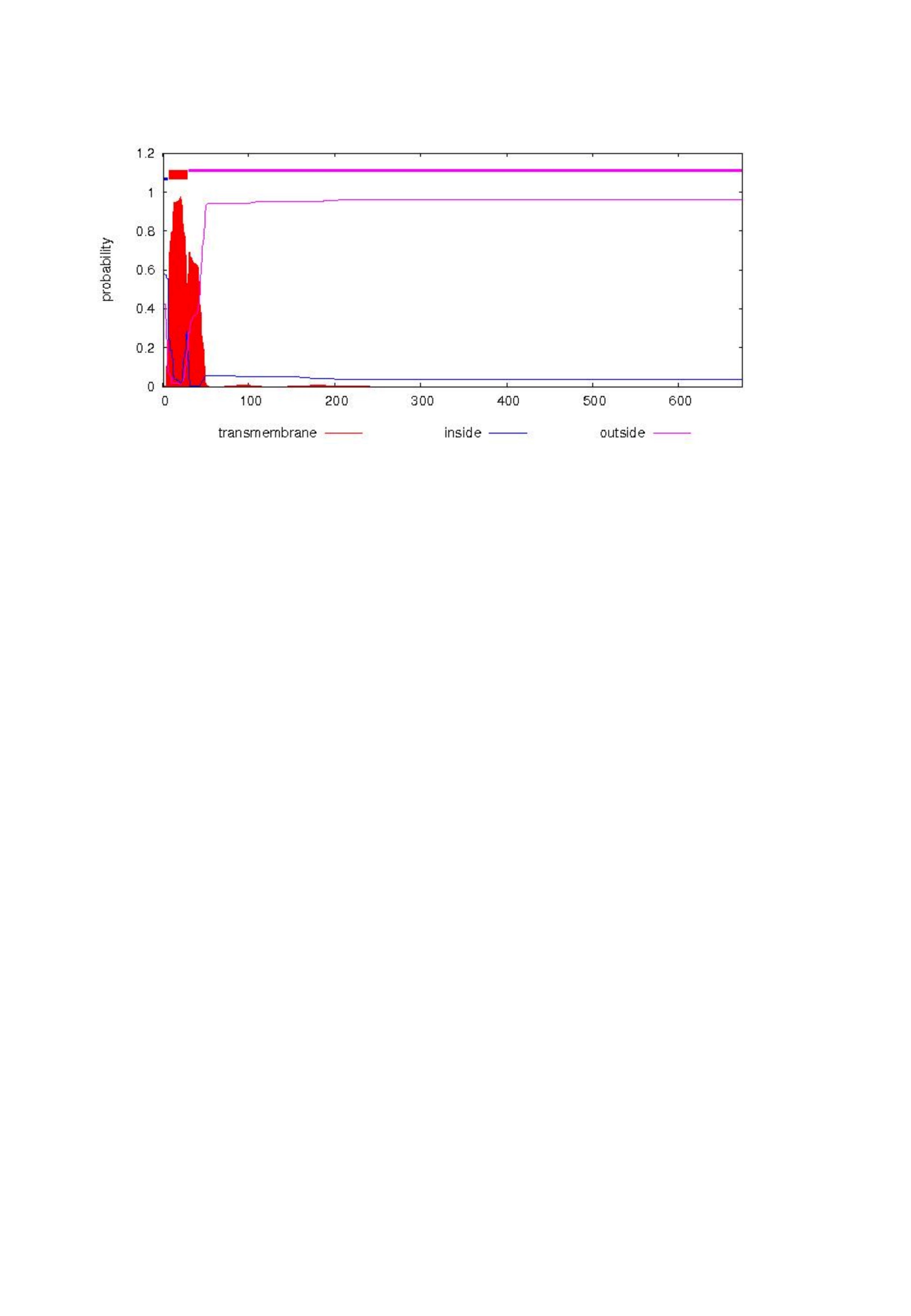


**Figure S13 Prediction of the transmembrane regions of Tn1a**

Topology prediction for Tn1a using a transmembrane domain hidden Markov model. (TMHMM version 2.0).


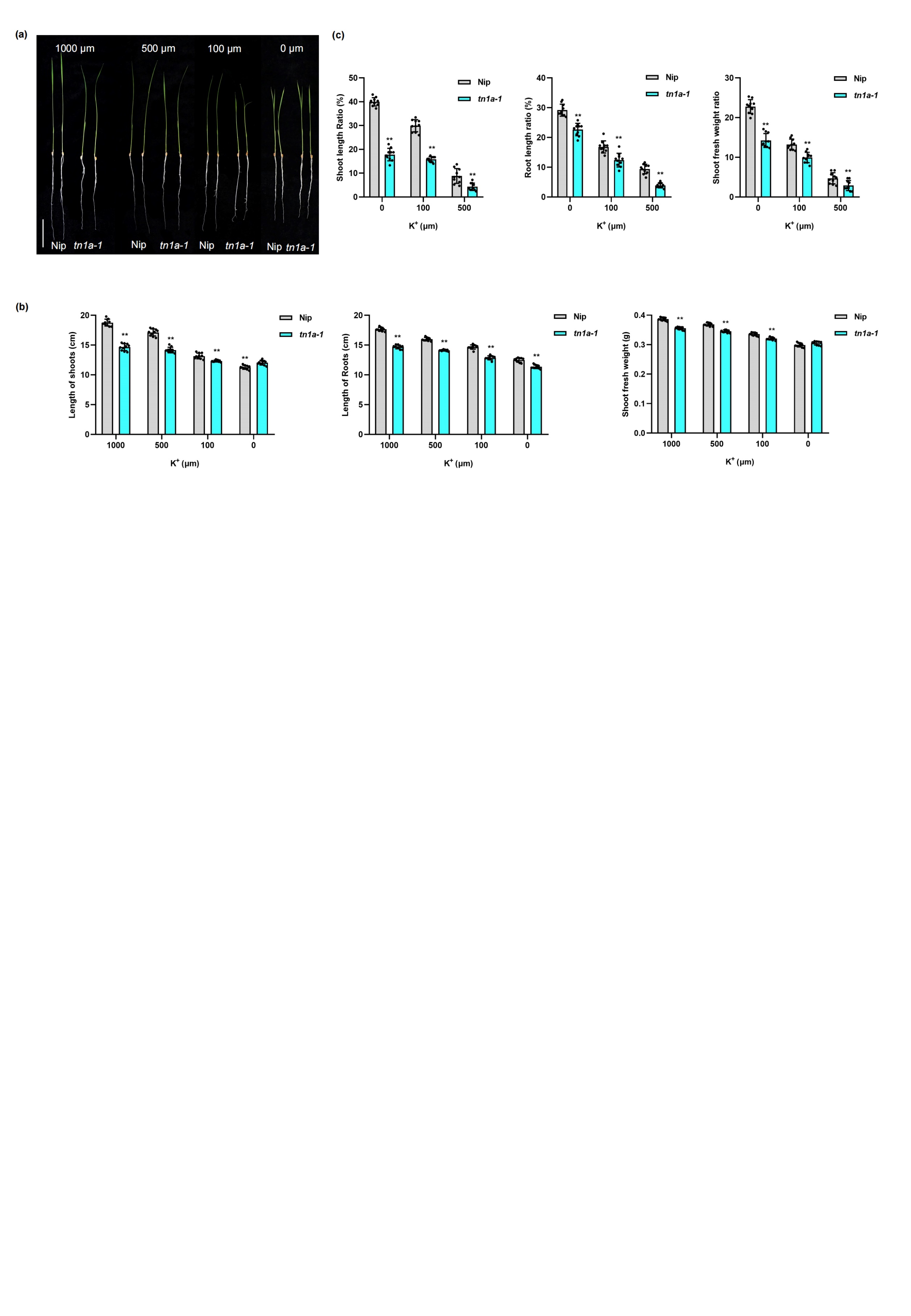


**Figure S14 *Tn1a* negatively regulates potassium deficiency stress in rice seedings**

(a) Performance of seedlings of Nip and *tn1a-1* grown for ten days in a hydroponic medium containing K^+^ as the concentrations indicated. Scale bar = 5 cm. (b) Shoot length, root length and shoot fresh weight of Nip and *tn1a-1* seedings shown in (a). (c) Shoot length ratio, root length ratio and shoot fresh weight ratio of Nip and *tn1a-1* seedings shown in (a). Shoot length ratio indicates the proportion of shoot length decreased under each K^+^ concentration compared to that under 1000 µm K^+^ condition, and root length ratio and shoot fresh weight ratio are calculated similarly. Values in (b-c) are mean ± SD (n=10), and statistical significance was determined using a two-sided *t*-test (***p* < 0.01).


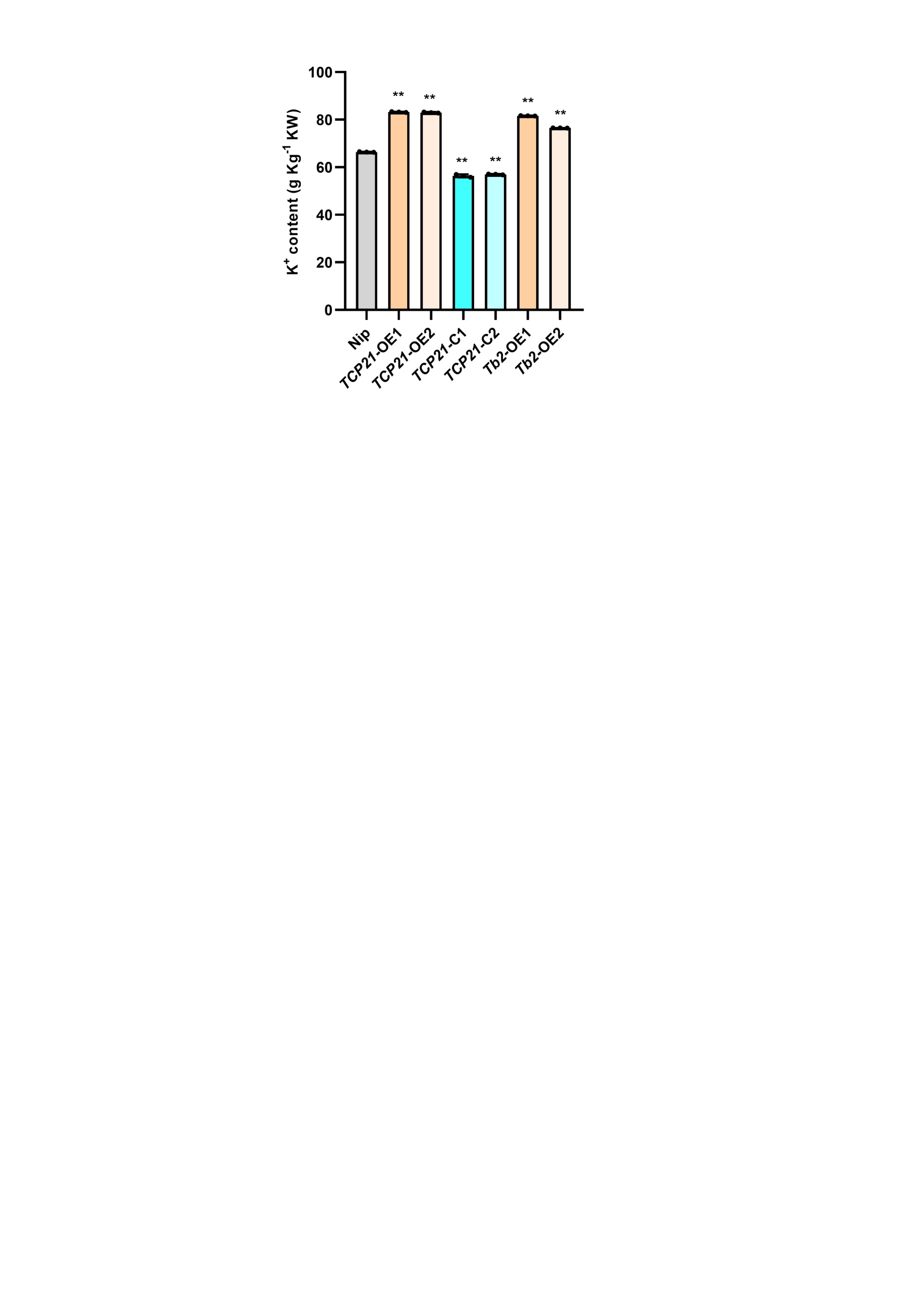


**Figure S15 *TCP21* and *Tb2* positively regulate plant K^+^ content**

K^+^ content in the shoots of Nip, *TCP21* overexpression and knockout lines and *Tb2* overexpression lines. Data are presented as mean ± SD (n = 3). *P*-values were determined using a two-sided Student’s *t*-test (***p* < 0.01).


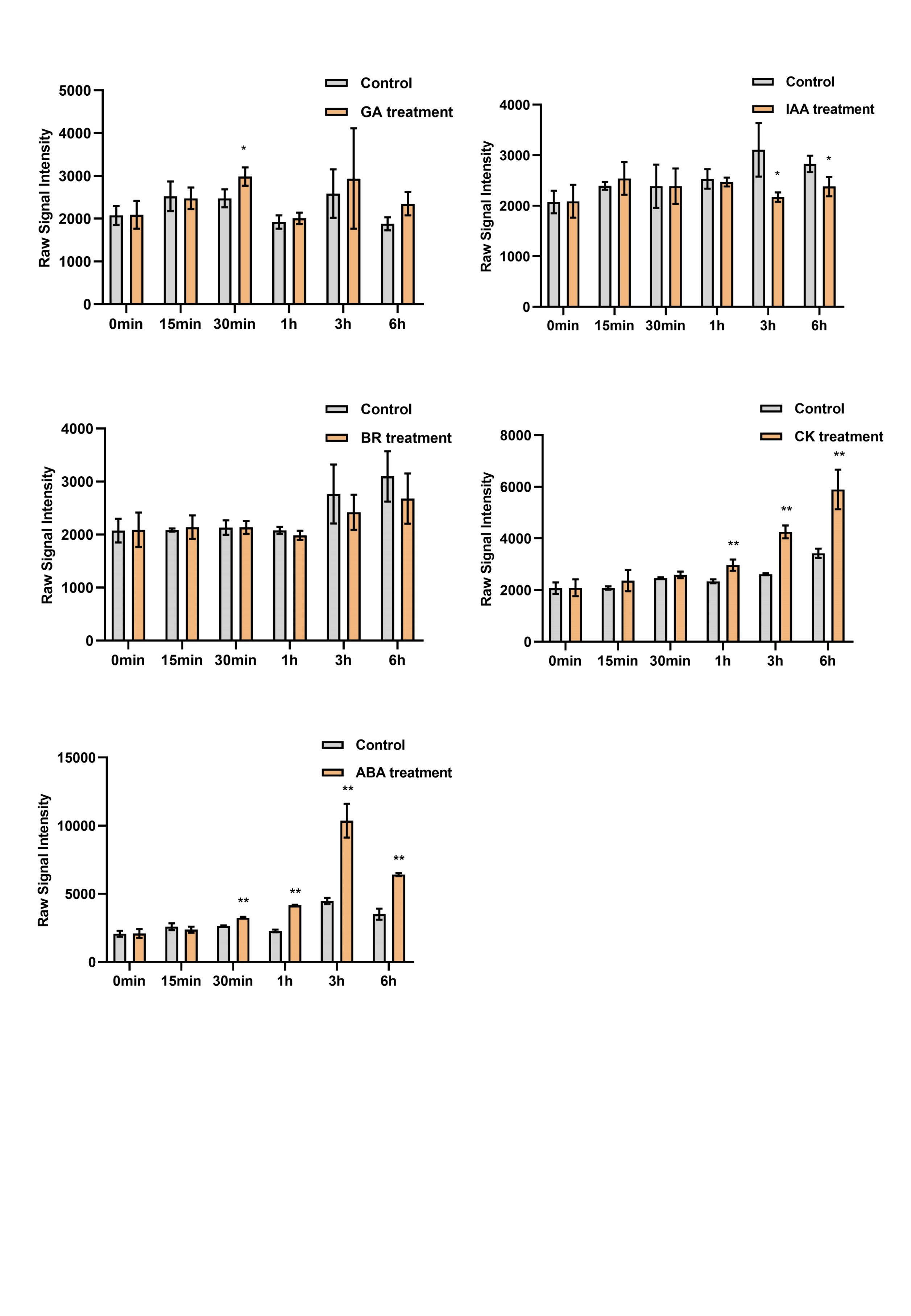


**Figure S16 The expression profile of *Tn1a* in root from rice seedlings treated with phytohormones**

Seeds of Nip were germinated and grown hydroponically in a growth chamber at 28ºC under continuous light. Seven-day old seedlings were transferred in culture solution containing the hormone (GA, IAA, BR, CK and ABA) and in culture solution without hormone to serve as control (mock treatment). Samples were collected after 15 min, 30 min, 1 h, 3 h and 6 h incubation for root with three replicates. Data were derived from RiceXpro (https://ricexpro.dna.affrc.go.jp/). Statistical significance was determined using a two- sided *t*-test (**p* < 0.05, ***p* < 0.01).


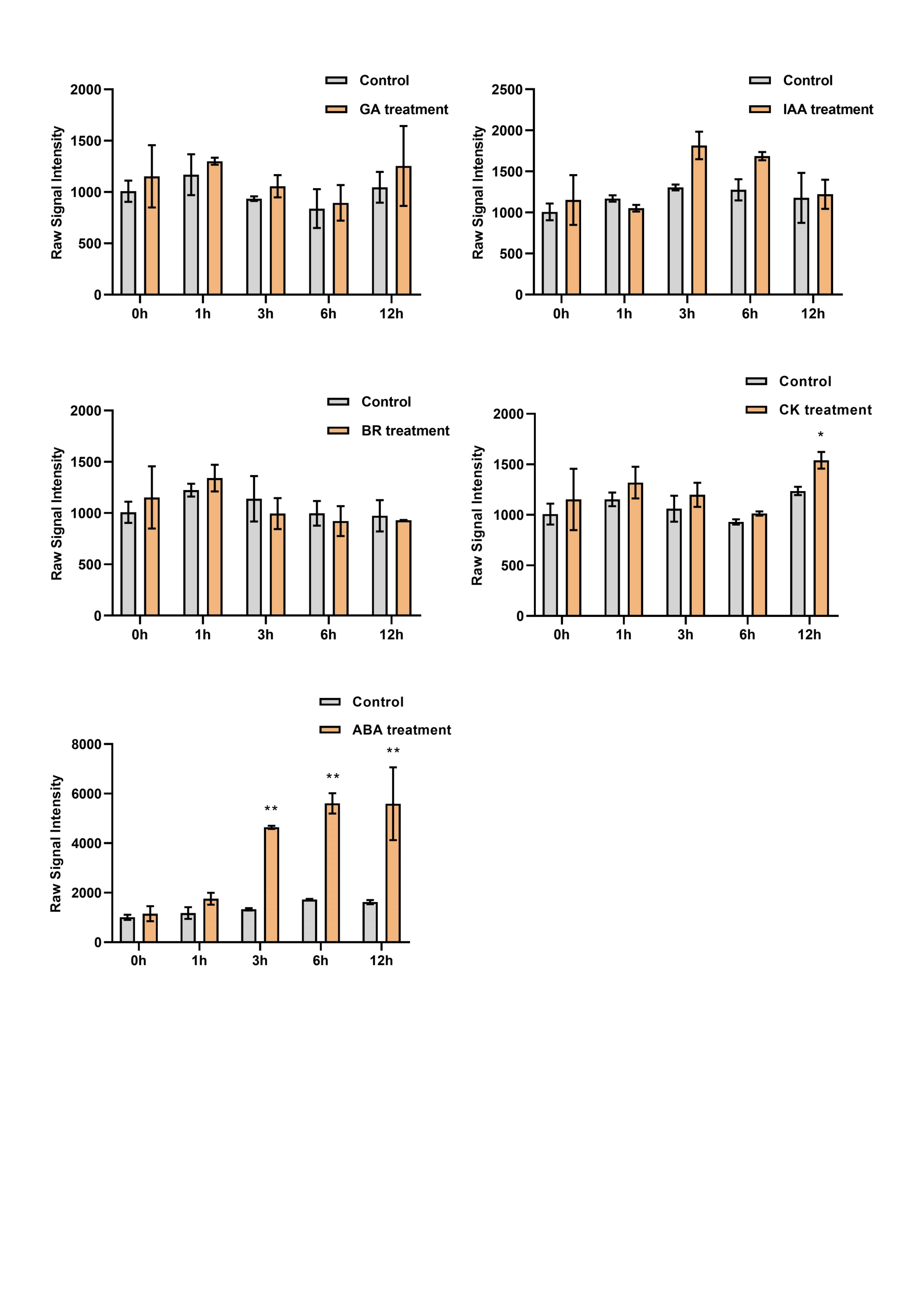


**Figure S17 The expression profile of *Tn1a* in shoot from rice seedlings treated with phytohormones**

Seeds of Nip were germinated and grown hydroponically in a growth chamber at 28ºC under continuous light. Seven-day old seedlings were transferred in culture solution containing the hormone (GA, IAA, BR, CK and ABA) and in culture solution without hormone to serve as control (mock treatment). Samples were collected after 1 h, 3 h, 6 h and 12 h incubation for shoot with two replicates. Data were derived from RiceXpro (https://ricexpro.dna.affrc.go.jp/). Statistical significance was determined using a two- sided *t*-test (**p* < 0.05, ***p* < 0.01).


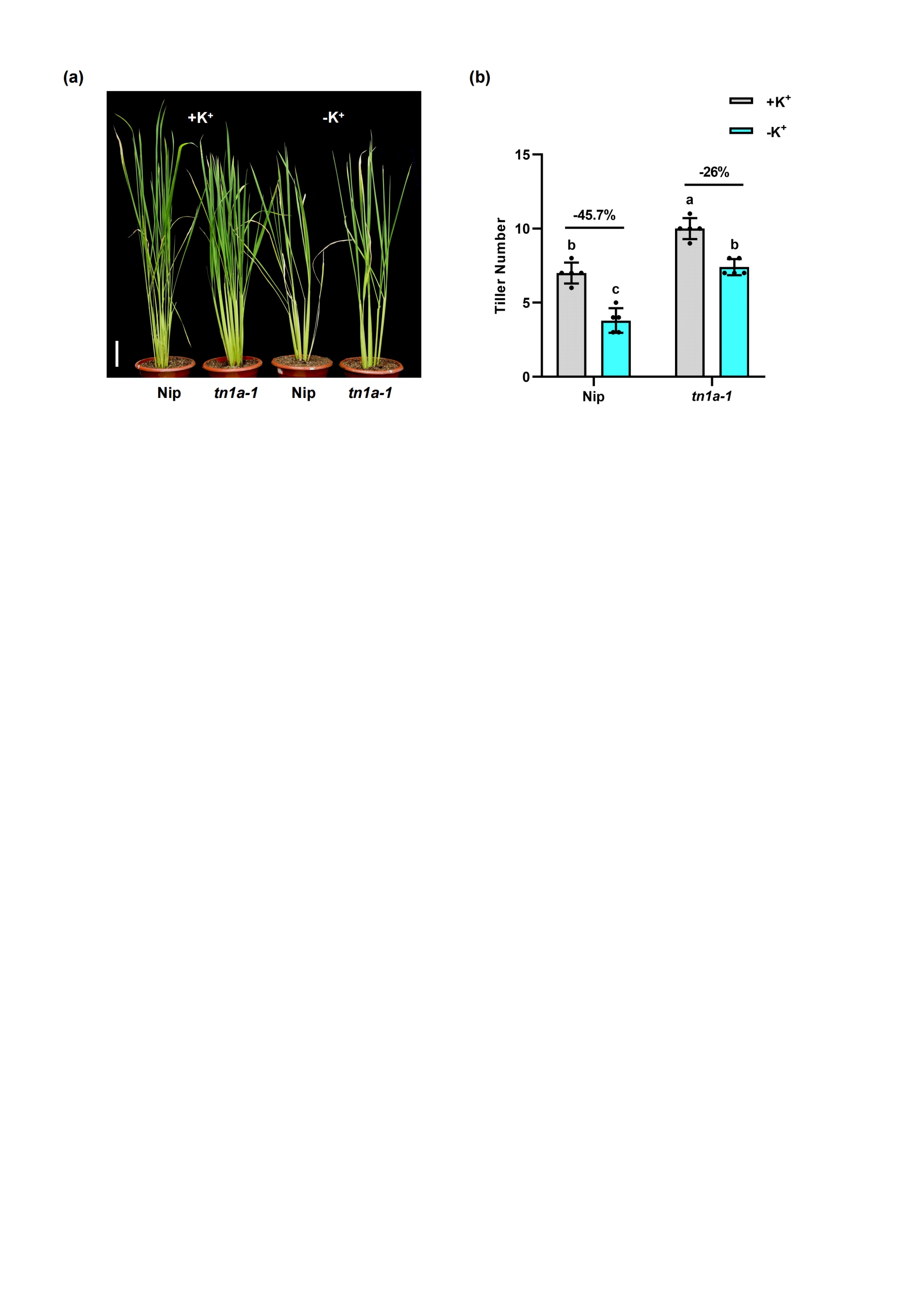


**Figure S18 *Tn1a* knockout promotes tiller number in K^+^ deficient soils**

(a) Plant architecture of Nip and knockout mutant *tn1a-1* in normal and K^+^ deficient soils. Scale bar = 10 cm. (b) Tiller numbers of Nip and *tn1a-1* shown in (a). Data are presented as mean ± SD (n = 5). Different letters indicate significant differences at *P* < 0.05 according to the two-sided Student’s *t*-test.


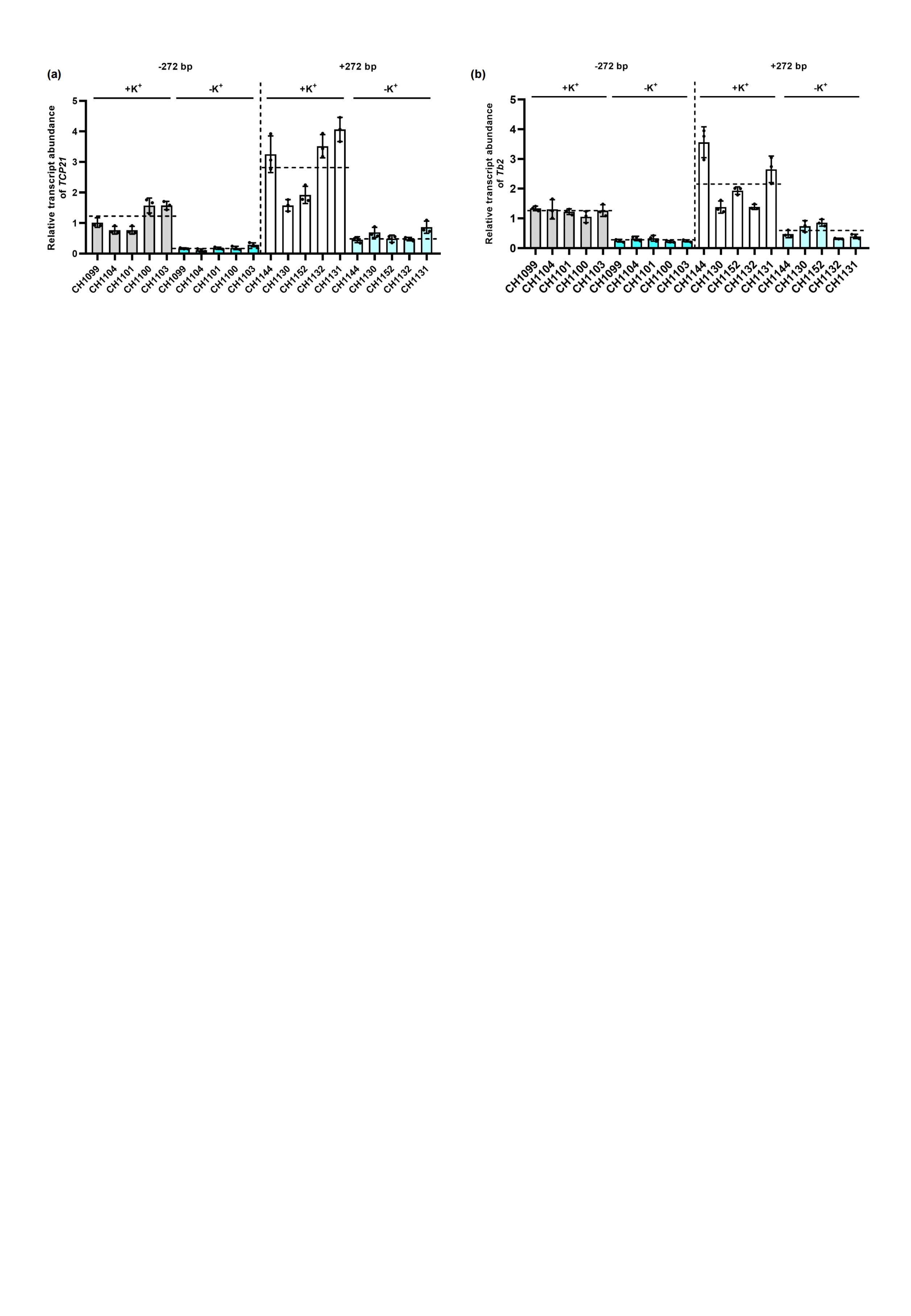


**Figure S19 *TCP21* and *Tb2* expression in *indica* subpopulation grouped by the 272 bp indel under normal K^+^ (1000 μmol) and K^+^ deficient (0 μmol) conditions.**


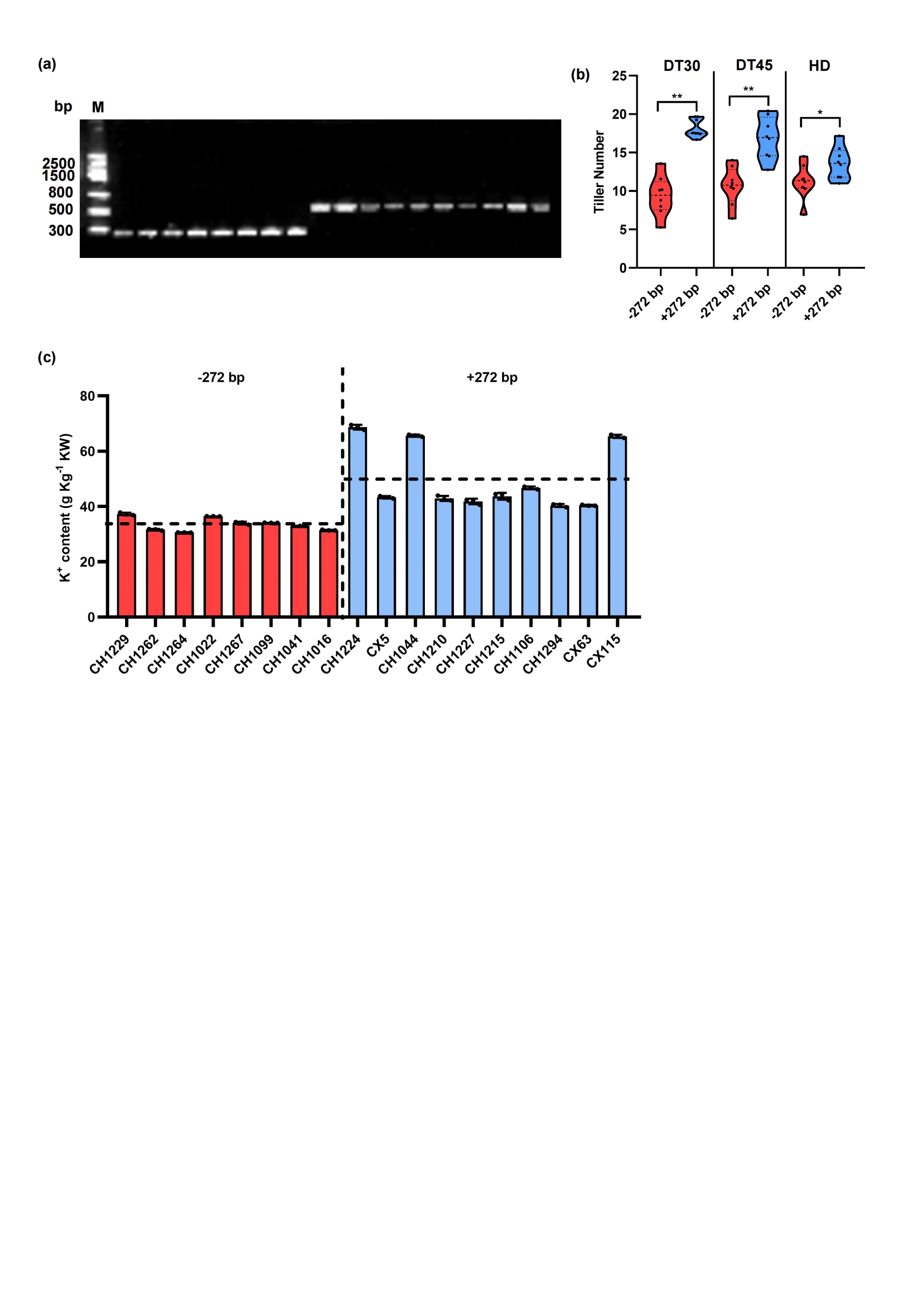


**Figure S20 Breeding utilization of 272 bp indel as a molecular marker**

(a) Genotyping analyses of 272 bp insertion/deletion in 18 diverse *indica* accessions using a pair of primers (Table S6) to amplify the 272 bp segment. (b) Tiller number of the 18 cultivated rice accessions in DT30, DT45, and HD stage. Statistical significance was determined using a two- sided *t*-test (**p* < 0.05, ***p* < 0.01). (c) K^+^ content in seedings of 18 cultivated rice accessions.
